# Supplementary material for: Which clinical research questions are the most important? Development and preliminary validation of the Australia & New Zealand Musculoskeletal (ANZMUSC) Clinical Trials Network Research Question Importance Tool (ANZMUSC-RQIT)
Source: PLoS One. 2023 Mar 17;18(3):e0281308. doi: 10.1371/journal.pone.0281308 (PMC10022765; doi:10.1371/journal.pone.0281308)
Supplement: S1 Appendix — (PDF) [file pone.0281308.s001.pdf]

Appendix 1 – novel best-worst scaling technique

This Appendix presents the experimental method of estimating the level of merit of a research proposal. The technique of analysis appears novel, and so it requires detailed description.

Formulation

Each research proposal is understood to possess different levels of five different dimensions, and the relative importance of the dimension-level combinations are to be assessed. This would enable an estimate of ‘utility’ to be calculated for any profile. The resulting estimate is understood to be an estimate of a true value that would be found if there were no sampling error or measurement error. So, there is to be an estimate of the reliability of the estimate, as usual, expressed as a ‘standard error’.

To provide a clear point of connection with the literature of ‘best-worst scaling’, (see below), this appendix uses the term ‘attribute’ instead of ‘dimension’. An attribute is represented using a letter A, B, C, D or E and a level is represented using a number 1,2,3, … The letters are nominal data: there is no concept of ordering; A is neither more important nor less important than B. The numbers are ordinal data: for any fixed attribute, a higher number is known to be associated with greater merit. However, the ordering only exists within levels for the same attribute, e.g. A2 is assumed to be more meritorious than A1, but A2 is not assumed to be more meritorious than B1. Also, the numbers are not interval data: for any attribute, the difference in merit between levels that are represented by 1 and 2 is not assumed to be the same as the difference in merit between levels 2 and 3. Likewise, the difference between levels 1 and 2 is not assumed to be the same for attribute A as for attribute B. These assumptions or stipulations form the basis of the technique.

An element is a combination of an attribute and a level, e.g. the element A3. For attributes A, C and E, the possible levels are 1, 2, 3 and 4, while for attributes B and D, the possible levels are 1, 2 and 3. So there are 18 possible elements, A1, A2, …, D4. An option is a pair of elements, e.g. (A3, B2). An individual choice-task is the simultaneous subjective ranking of three and only three options into 'best', 'worst' and by implication 'middle'. Ranking the options as being of equal importance is not permitted. The set of choice-tasks (or a subset of them) is given to respondents who represent the relevant population of respondents. The data and the model are then used to estimate the utilities (merits) of the 18 elements.

A profile is a set of elements, one for each attribute, e.g. (A3, B2, C1, D1, E3). The merit of a research proposal is represented by the utility of the corresponding profile, which is the sum of the utilities of the elements. The objective is to represent the utilities of all the possible profiles on a logical and useful scale. These utilities are estimated by scores, the reliabilities of which will be described by their standard errors. This formulation enables an analysis using the principles of frequentist, classical, statistics.

Design and model

Each choice task involves comparing three options that feature the same pair of dimensions, for example, the options (A2, C3), (A3, C2) and (A4, C1). According to the assumptions of the previous steps in the project, the levels for any attribute are correctly ordered. This meant that, except for checking these assumptions or estimating the variances of the measurement errors, there is nothing to be gained in an implied comparison such as (A2, C3) vs (A1, C2), where the levels in the first option are both greater than the corresponding levels in the second option (one option would be said to 'dominate' the other

option). Choice-tasks involving such comparisons were excluded to avoid wasting resources. An exhaustive analysis of the remaining possibilities then led to the identification of 86 different possible choice-tasks. For simplicity and economy, respondents were randomized to one of three sets of 30 choice tasks, each set having 2 choice-tasks in common. As a result, each option was not assessed an equal number of times. To some extent, this could have been avoided with block-randomization, but with an online survey this was not trivial.

The next step in the procedure is to propose a model for the respondent's method of choosing the 'best' and 'worst' among three options. The model adopted is the standard Thurstone model (16), where the reaction of a person to a stimulus (an option) is given by the size of the stimulus (the sum of the utilities of the two elements in the option), plus normally distributed random measurement error. (The assumption that the error is from a logistic distribution instead, which corresponds to the Bradley-Terry model of choice (19, 20), would make very little difference in practice. The normal model seems preferable because of the principle underlying the central limit theorem.) The fact that there are many implied comparisons in any individual choice-task means that the three effective measurement errors in the Thurstone model must be viewed as having arisen from a trivariate normal distribution.

Model 1, the full model, is as follows: Let *a* and *b* represent letters (attributes), say A and C, while *i* and *j* represent numbers (levels), and let $\theta_{a_i}$and θ_{b_j} be the utilities of the elements a_i and b_j. In a choice-task at time *t* involving an option (a_i, b_j), respondent *k* has a subjective reaction to this option given by $Y_{kta_ib_j} = \theta_{a_i} + \theta_{b_j} + \epsilon_{kta_ib_j}$ where $\epsilon_{kta_ib_j}$ is a normally distributed error with mean 0 and variance σ_k^2 . Each $\epsilon_{kta_ib_j}$ is independent. Respondent *k* rates option (a_{i1}, b_{j1}) as being more meritorious than option (a_{i2}, b_{j2}) at

time *t* if $Y_{kta_{i1}b_{j1}} > Y_{kta_{i2}b_{j2}}$. The error model is 'multivariate' in that, in the choice-task at time *t*, an error such as $\epsilon_{kta_{i1}b_{j1}}$ affects the comparison of option (a_{i1}, b_{j1}) with option (a_{i2}, b_{j2}) and also the comparison of option (a_{i1}, b_{j1}) with option (a_{i3}, b_{j3}). The time variable *t* simply represents the occasion (choice-task) on which the option is presented to the respondent. The inclusion of the variable *t* in the description simplifies the model: it avoids the need to link the errors in different choice-tasks involving the same option. Model 2, which is represented equivalently in the text by Model 2a and Model2b, is the same as Model 1 except that it constrains the variances σ_k^2 to be equal for each respondent (21, 22).

Best-worst scaling

This method of estimating the merits of different entities can be considered as a method of `best-worst scaling', which is a model that generalizes the standard idea of an individual 'paired-comparison' to the simultaneous comparison of more than two entities. However, this implementation of the best-worst idea appears to be novel. Louviere, Flynn and Marley have developed the idea of best-worst scaling, and they identify three different types, which they call Case I (the 'object case'), Case 2 (the 'profile case') and Case 3 (the 'multi-profile' case) (23).

In Case I, 'objects' that are isolated and whole within themselves, such as individual candidates in an election, are compared. In our situation, an object would be an element, such as A1, which is an indivisible unit: the entity in our description would be an element. However, our description involves the comparison of different options, (i.e. pairs of elements), so our method is not Case 1 best-worst scaling. In Case 2, 'profiles' analogous to our profiles are compared, perhaps in a full factorial design to explore all possible profiles at once. However, our method does not compare full profiles: it compares different

possibilities for parts of profiles, these being our options. In Case 3, three or more profiles are compared simultaneously. Thus, our context of comparing and ranking differs from the three basic contexts described by Louviere, Flynn and Marley. Our method might be said to reflect `Case 4 best-worst-scaling (the sub-profile case)'.
