# Supplementary material for: Which clinical research questions are the most important? Development and preliminary validation of the Australia & New Zealand Musculoskeletal (ANZMUSC) Clinical Trials Network Research Question Importance Tool (ANZMUSC-RQIT)
Source: PLoS One. 2023 Mar 17;18(3):e0281308. doi: 10.1371/journal.pone.0281308 (PMC10022765; doi:10.1371/journal.pone.0281308)
Supplement: S2 Appendix — (PDF) [file pone.0281308.s002.pdf]

Appendix 2 – the hypothetical research questions used to evaluation the reliability of RQIT

Research question template

The research question is:

How does diet effect gout?

Please tick one box for each of the following categories:

Category	Content relevant to this research question	Your rating of this category
Extent to which the question is important to patients and other health decision-makers	Patient's understand the importance of dietary changes to reduce their gout symptoms; however, they regularly feel that dietary advice from their GP's is unclear and leaves them confused (Liddle et al., 2017). In priority setting exercises, patients identify that better understanding of dietary implications is a primary concern (Singh, 2014). A lack of robust clinical trials examining the impact of diet (Moi, Sriranganathan, Edwards, & Buchbinder, 2013) and varying authoritative guidelines for dietary management, creates difficulties for clinicians and justifies the need for research (Stamp, 2017).	☐ Not shown to be important to either patients or decision-makers<br>☐ Shown to be important to health decision-makers but not patients<br>☐ Shown to be important to patients but not decision-makers<br>☐ Shown to be important to both patients and decision-makers
That it addresses an area of high patient burden	Gout symptoms range in intensity and in some cases can be a debilitatingly painful condition that can severely impact activities of daily living, requiring care from family and resulting in an inability to work during flair-ups. In Australasia, gout attributed to 3088 DALY's (Smith et al., 2014) and is often poorly diagnosed and treated. Symptoms and appearance are often embarrassing to patients which creates isolation and depression in individuals (Lindsay, Gow, Vanderpyl, Logo, & Dalbeth, 2011; Martini et al., 2012).	☐ Mild symptoms and little or no associated disability<br>☐ Moderate symptoms and some disability<br>☐ Significantly disabling, associated with mortality risk or no effective treatments available
That it addresses an area of high social burden	Global prevalence of gout is climbing and was estimated to be 0.08% worldwide (Smith et al., 2014), although this is substantially higher within New Zealand and Australia. Calculated estimates for New Zealand are between 2.69% and 2.89% for all ages, and as high as 41% in some subpopulations (Winnard et al., 2012).	☐ Condition is rare<br>☐ Condition is somewhat common<br>☐ Condition is common<br>☐ Condition is highly prevalent

Potential reduction in patient and/or social burden due to (clinical or implementation) intervention	Gout occurs as a result of hyperuricemia due to improper purine metabolism and reduced uric acid excretion at the kidneys. Elimination of some foods are likely to prevent hyperuricemia (Yu, See, Huang, Yang, & Sun, 2008), however the relationship between chronic kidney disease and serum uric acid is poorly understood. It is likely that any changes in gout presentation as a result of dietary interventions will not fundamentally alter the disease.	☐ Symptomatic treatment only and small potential effect size<br>☐ Symptomatic treatment and large potential effect size<br>☐ Potential for intervention to treat both symptoms and underlying disease pathology<br>☐ Potential for cure or fundamental alteration of disease course
Potential scalability and uptake or intervention	Dietary interventions typically have poor uptake and adherence by patients. Attrition and adherence are often impacted by a lack of immediate results and by the burden of lifestyle change, particularly with strict diets of reduced variety (Crichton et al., 2012). While well researched dietary interventions for gout exist and could be scaled through simple training at a clinician level, good uptake requires a high level of patient support, education, community engagement, and follow up contact to ensure patients maintain any program. Special challenges exist around cultural approaches to diet and meal sharing, especially in Pacific Island and Māori communities (Martini et al., 2012).	☐ Low potential for scalability and uptake<br>☐ High potential for uptake but low scalability<br>☐ High potential for scalability but low potential for uptake<br>☐ High potential for both scalability and uptake
Extent to which the question addresses health equity	The risk of developing gout is higher within indigenous Australian and New Zealand populations. Prevalence within Australian aboriginal and males has risen from 0% in 1965 to 9.6% in 2002 (Robinson, Taylor, & Merriman, 2012). NZ Māori prevalence is 6.06%, with elderly Māori males living in the areas of highest deprivation experiencing prevalence greater than 40% (Winnard et al., 2012). Changes to diet since colonialization are likely to have played a part in the development of gout in indigenous populations and a better understanding is warranted as to how diet impacts this disease.	☐ No attempt to address health equity<br>☐ Discussed using Progress Plus items (O'Neill et al., 2014) but intervention not relevant or appropriate<br>☐ Intervention shown to have some potential application to improving health equity issues<br>☐ The intervention is explicitly designed to improve health equity issues

Final question:

In your opinion, do you think this research question is important enough for ANZMUSC to endorse it (assuming all other endorsement criteria are met)?

☐ Yes

☐ No

References

Crichton, G. E., Howe, P. R. C., Buckley, J. D., Coates, A. M., Murphy, K. J., & Bryan, J. (2012). Long-term dietary intervention trials: critical issues and challenges. *Trials*, *13*(1), 111. https://doi.org/10.1186/1745-6215-13-111

Liddle, J., Richardson, J. C., Mallen, C. D., Hider, S. L., Chandratre, P., & Roddy, E. (2017). 181. I FOUND IT MOST CONFUSING, I DIDN'T KNOW WHAT TO BELIEVE: EXPLORING PATIENT DECISION MAKING AROUND GOUT AND DIET. *Rheumatology*, *56*(suppl_2). https://doi.org/10.1093/rheumatology/kex062.182

Lindsay, K., Gow, P., Vanderpyl, J., Logo, P., & Dalbeth, N. (2011). The experience and impact of living with gout: A study of men with chronic gout using a qualitative grounded theory approach. *Journal of Clinical Rheumatology*, *17*(1), 1–6. https://doi.org/10.1097/RHU.0b013e318204a8f9

Martini, N., Bryant, L., Karu, L. Te, Aho, L., Chan, R., Miao, J., … Tieu, A. (2012). Living with gout in New Zealand: An exploratory study into People's knowledge about the disease and its treatment. *Journal of Clinical Rheumatology*, *18*(3), 125–129. https://doi.org/10.1097/RHU.0b013e31824e1f6f

Moi, J. H., Sriranganathan, M. K., Edwards, C. J., & Buchbinder, R. (2013). Lifestyle interventions for chronic gout. *Cochrane Database of Systematic Reviews*, (September), 2010–2010. https://doi.org/10.1002/14651858.CD010039.pub2

Robinson, P. C., Taylor, W. J., & Merriman, T. R. (2012). Systematic review of the prevalence of gout and hyperuricaemia in Australia. *Internal Medicine Journal*, *42*(9), 997–1007. https://doi.org/10.1111/j.1445-5994.2012.02794.x

Singh, J. A. (2014). Research Priorities in Gout: The Patient Perspective. *The Journal of Rheumatology*, *41*(3), 615–616. https://doi.org/10.3899/jrheum.131258

Smith, E., Hoy, D., Cross, M., Merriman, T. R., Vos, T., Buchbinder, R., … March, L. (2014). The global burden of gout: Estimates from the Global Burden of Disease 2010 study. *Annals of the Rheumatic Diseases*, *73*(8), 1470–1476. https://doi.org/10.1136/annrheumdis-2013-204647

Stamp, L. K. (2017). Major unanswered questions in the clinical gout field. *Current Opinion in Rheumatology*, *29*(2), 171–177. https://doi.org/10.1097/BOR.0000000000000367

Winnard, D., Wright, C., Taylor, W. J., Jackson, G., Te Karu, L., Gow, P. J., … Dalbeth, N. (2012). National prevalence of gout derived from administrative health data in Aotearoa New Zealand. *Rheumatology*, *51*(5), 901–909. https://doi.org/10.1093/rheumatology/ker361

Yu, K.-H., See, L.-C., Huang, Y.-C., Yang, C.-H., & Sun, J.-H. (2008). Dietary Factors Associated with Hyperuricemia in Adults. *Seminars in Arthritis and Rheumatism*, *37*(4), 243–250. https://doi.org/10.1016/J.SEMARTHRIT.2007.04.007

Research question template

The research question is:

What is the comparative effectiveness of 4 combination pharmacological and non-pharmacological management strategies for pain and function in patients with fibromyalgia?

A: CBT + self-management education programme + placebo vs.

B: Sequential pregabalin, duloxetine, combination pharmacological therapy vs.

C: CBT + self-management + sequential pharmacotherapy vs.

D: Graded exercise + CBT + self-management + sequential pharmacotherapy

Please tick one box for each of the following categories:

Category	Content relevant to this research question	Your rating of this category
Extent to which the question is important to patients and other health decision-makers	Clinicians and patients share a number of priorities regarding the management of fibromyalgia: pain, cognitive function, fatigue, and emotional distress (P. J. Mease et al., 2008). Finding an effective combination therapy that impacts each of these domains would likely assist patients with their symptoms and allow clinicians to provide better support, education and care.	☐ Not shown to be important to either patients or decision-makers<br>☐ Shown to be important to health decision-makers but not patients<br>☐ Shown to be important to patients but not decision-makers<br>☐ Shown to be important to both patients and decision-makers
That it addresses an area of high patient burden	Fibromyalgia is a poorly understood condition that likely results from central sentisiation and produces a hypersentitivity to touch stimulus resulting in a widespread chronic pain comparable to rheumatoid arthritis and a fatigue state (P. Mease, 2005). Patients often experience emotional disturbances, poor sleep, headaches and irritable bowel syndrome. Fibromyalgia is significantly disabling with 30% of patients reporting loss of employment and 15% reporting impact to physical activity (Greenfield, Fitzcharles, & Esdaile, 1992).	☐ Mild symptoms and little or no associated disability<br>☐ Moderate symptoms and some disability<br>☐ Significantly disabling, associated with mortality risk or no effective treatments available

That it addresses an area of high social burden	Worldwide prevalence for fibromyalgia ranges from 0.4% to 11% (Marques, Santo, Berssaneti, Matsutani, & Yuan, 2017; Queiroz, 2013). The condition is twice as likely to affect women as men. No prevalence data exists for Oceania.	☐ Condition is rare<br>☐ Condition is somewhat common<br>☐ Condition is common<br>☐ Condition is highly prevalent
Potential reduction in patient and/or social burden due to (clinical or implementation) intervention	Level 1A evidence exists for the use of exercise, CBT, and pharmacological monotherapies in the management of fibromyalgia (Clauw, 2014), however few studies have examined the effectiveness of combination therapies. The exact cause of fibromyalgia is still unknown, however evidence suggests that CBT might positively affect the emotional and cognitive aspects of the disease, exercise on the musculoskeletal components of pain sensitisation, and combination pharmacotherapy to reduce pain (Bernardy, Klose, Busch, Choy, & Häuser, 2013; Busch, Barber, Overend, Peloso, & Schachter, 2007; Thorpe, Shum, Moore, Wiffen, & Gilron, 2018).	☐ Symptomatic treatment only and small potential effect size<br>☐ Symptomatic treatment and large potential effect size<br>☐ Potential for intervention to treat both symptoms and underlying disease pathology<br>☐ Potential for cure or fundamental alteration of disease course
Potential scalability and uptake or intervention	The management of combination therapy would require coordination between several professional modalities and therefore complex in organisation and scalability. Exercise therapies require considerable patient motivation and patients with fibromyalgia may show hesitance without the confidence that exercise will not create negative outcomes (Meyer & Lemley, 2000). Motivation is also a major cause of withdrawal from CBT for patients with fibromyalgia (Bernardy et al., 2013). Overcoming barriers for combination therapy including CBT or PA may be challenging an impact on programme uptake.	☐ Low potential for scalability and uptake<br>☐ High potential for uptake but low scalability<br>☐ High potential for scalability but low potential for uptake<br>☐ High potential for both scalability and uptake

Extent to which the question addresses health equity	Prevalence of fibromyalgia appears to be higher in patients with lower education and in those with lower household income (Queiroz, 2013). Data regarding prevalence amongst New Zealand Maori or Australian Aboriginal does not exist. 30% of patients with rheumatic conditions – such as osteoarthritis, rheumatoid arthritis or gout – meet the conditions for fibromyalgia (Clauw, 2014). Considering the high prevalence of rheumatic conditions in New Zealand Maori and Aboriginals (McDougall, Hurd, & Barnabe, 2017), it may be that a number of cases exist undiagnosed in these populations.	☐ No attempt to address health equity<br>☐ Discussed using Progress Plus items (O'Neill et al., 2014) but intervention not relevant or appropriate<br>☐ Intervention shown to have some potential application to improving health equity issues<br>☐ The intervention is explicitly designed to improve health equity issues

Final question:

In your opinion, do you think this research question is important enough for ANZMUSC to endorse it (assuming all other endorsement criteria are met)?

☐ Yes

☐ No

References

Bernardy, K., Klose, P., Busch, A. J., Choy, E. H., & Häuser, W. (2013). Cognitive behavioural therapies for fibromyalgia. *Cochrane Database of Systematic Reviews*, *2017*(6). https://doi.org/10.1002/14651858.CD009796.pub2

Busch, A. J., Barber, K. A. R., Overend, T. J., Peloso, P. M. J., & Schachter, C. L. (2007). Exercise for treating fibromyalgia syndrome. *Cochrane Database of Systematic Reviews*, (4). https://doi.org/10.1002/14651858.CD003786.pub2

Clauw, D. J. (2014). Fibromyalgia. *JAMA*, *311*(15), 1547. https://doi.org/10.1001/jama.2014.3266

Greenfield, S., Fitzcharles, M. -A, & Esdaile, J. M. (1992). Reactive fibromyalgia syndrome. *Arthritis & Rheumatism*, *35*(6), 678–681. https://doi.org/10.1016/j.ejcts.2005.09.008

Marques, A. P., Santo, A. de S. do E., Berssaneti, A. A., Matsutani, L. A., & Yuan, S. L. K. (2017). Prevalence of fibromyalgia: literature review update. *Revista Brasileira de Reumatologia (English Edition)*, *57*(4), 356–363. https://doi.org/10.1016/j.rbre.2017.01.005

McDougall, C., Hurd, K., & Barnabe, C. (2017). Systematic review of rheumatic disease epidemiology in the indigenous populations of Canada, the United States, Australia, and New Zealand. *Seminars in Arthritis and Rheumatism*, *46*(5), 675–686. https://doi.org/10.1016/J.SEMARTHRIT.2016.10.010

Mease, P. (2005). Fibromyalgia syndrome: review of clinical presentation, pathogenesis, outcome measures, and treatment. *The Journal of Rheumatology. Supplement*, *75*, 6–21. Retrieved from http://www.jrheum.org/content/75/6http://jrheum.com/faqwww.jrheum.orgwww.jrheum.orgDownloadedfrom

Mease, P. J., Arnold, L. M., Crofford, L. J., Williams, D. A., Russell, I. J., Humphrey, L., … Martin, S. A. (2008). Identifying the clinical domains of fibromyalgia: Contributions from clinician and patient delphi exercises. *Arthritis Care and Research*, *59*(7), 952–960. https://doi.org/10.1002/art.23826

Meyer, B. B., & Lemley, K. J. (2000). Utilizing exercise to affect the symptomology of fibromyalgia: a pilot study.

Medicine and Science in Sports and Exercise, *32*(10), 1691–1697. Retrieved from http://www.msse.org

Queiroz, L. P. (2013). Worldwide Epidemiology of Fibromyalgia. *Current Pain and Headache Reports*, *17*(8), 356. https://doi.org/10.1007/s11916-013-0356-5

Thorpe, J., Shum, B., Moore, R. A., Wiffen, P. J., & Gilron, I. (2018). Combination pharmacotherapy for the treatment of fibromyalgia in adults. *Cochrane Database of Systematic Reviews*, *2018*(2). https://doi.org/10.1002/14651858.CD010585.pub2

Research question template

The research question is:

Is a wrist splint better than corticosteroid injection for people with de Quervain's tenosynovitis in terms of pain and function at 3 months?

Please tick one box for each of the following categories:

Category	Content relevant to this research question	Your rating of this category
Extent to which the question is important to patients and other health decision-makers	Steroid injection for treatment of tendinopathies appears to be widely accepted by clinicians. For patients, there is some concern over the use of steroids and many prefer a more conservative approach which may be found using a wrist splint. In breastfeeding women, who have elevated risk of developing de Quervain's tenosynovitis, there is a perception of risk for their children associated with steroid use. A fear of needles is also a valid contraindication to corticosteroid injection that might warrant use of splint, particularly when heightened needle fear can create a negative feedback loop thereby creating anxiety and non-compliance (McMurtry et al., 2015).	☐ Not shown to be important to either patients or decision-makers<br>☐ Shown to be important to health decision-makers but not patients<br>☐ Shown to be important to patients but not decision-makers<br>☐ Shown to be important to both patients and decision-makers
That it addresses an area of high patient burden	Pain is activity dependent, felt over the distal radius, and can last several days. Symptoms are functionally limiting and can impact the ability to work or perform household or leisure activities. The pain is normally aggravated with ulnar deviation of the wrist and patients often complain of difficulty opening jars, wringing a washcloth or playing golf (Goel & Abzug, 2015).	☐ Mild symptoms and little or no associated disability<br>☐ Moderate symptoms and some disability<br>☐ Significantly disabling, associated with mortality risk or no effective treatments available
That it addresses an area of high social burden	de Quervain's tenosynovitis is common with prevalence of 0.5% in men and 1.3% in women (Walker-Bone, Palmer, Reading, Coggon, & Cooper, 2004). There is elevated occurrence in postpartum women (Johnson, 1991) and it is possible that a link to phone usage may see a new younger generation of sufferers (Ali et al., 2014).	☐ Condition is rare<br>☐ Condition is somewhat common<br>☐ Condition is common<br>☐ Condition is highly prevalent

Potential reduction in patient and/or social burden due to (clinical or implementation) intervention	Although termed as tenosynovitis, the cause of injury is a degenerative thickening of the tendon as it enters the synovial sheath and not an inflammatory process (Clarke, Lyall, Grant, & Matthewson, 1998). While immobilisation may reduce symptoms, it seems unlikely that this would be sufficient to reverse the pathological process.	☐ Symptomatic treatment only and small potential effect size<br>☐ Symptomatic treatment and large potential effect size<br>☐ Potential for intervention to treat both symptoms and underlying disease pathology<br>☐ Potential for cure or fundamental alteration of disease course
Potential scalability and uptake or intervention	In a review of treatment options for de Quervain's tenosynovitis it was shown that, although there is a relative dearth of quality research, steroidal injections appear to be highly effective (Peters-Veluthamaningal, Van Der Windt, Winters, & Meyboom-de Jong, 2009). With this said, there are relative risks associated with steroid use and effectiveness is dependent on accurate delivery in to the small synovial sheath.<br><br>Splinting is a simple, cost-effective mechanism for managing injury that requires a comparably low level of training and could be easily administered by a range of health providers including nurses, physiotherapists and occupational therapists.	☐ Low potential for scalability and uptake<br>☐ High potential for uptake but low scalability<br>☐ High potential for scalability but low potential for uptake<br>☐ High potential for both scalability and uptake
Extent to which the question addresses health equity	Very little is known as to how prevalence of de Quervain's differs or impacts those of from different cultures, socio-economic regions, education status, or physical locations. More research is required.	☐ No attempt to address health equity<br>☐ Discussed using Progress Plus items (O'Neill et al., 2014) but intervention not relevant or appropriate<br>☐ Intervention shown to have some potential application to improving health equity issues<br>☐ The intervention is explicitly designed to improve health equity issues

Final question:

In your opinion, do you think this research question is important enough for ANZMUSC to endorse it (assuming all other endorsement criteria are met)?

☐ Yes

☐ No

References

Ali, M., Asim, M., Danish, S. H., Ahmad, F., Iqbal, A., & Hasan, S. D. (2014). Frequency of De Quervain's tenosynovitis and its association with SMS texting. *Muscles, Ligaments and Tendons Journal*, *4*(1), 74–78. Retrieved from http://www.ncbi.nlm.nih.gov/pubmed/24932451

Clarke, M. T., Lyall, H. A., Grant, J. W., & Matthewson, M. H. (1998). The histopathology of de Quervain's disease. *Journal of Hand Surgery*, *23*(6), 732–734. https://doi.org/10.1016/S0266-7681(98)80085-5

Goel, R., & Abzug, J. M. (2015). de Quervain's tenosynovitis: a review of the rehabilitative options. *Hand*, *10*(1), 1–5. https://doi.org/10.1007/s11552-014-9649-3

Johnson, C. A. (1991). Occurrence of de Quervain's disease in postpartum women. *The Journal of Family Practice*, *32*(3), 325–327.

McMurtry, C. M., Riddell, R. P., Taddio, A., Racine, N., Asmundson, G. J. G., Noel, M., … Shah, V. (2015). Far from "just a poke": Common painful needle procedures and the development of needle fear. *Clinical Journal of Pain*, *31*(10), S3–S11. https://doi.org/10.1097/AJP.0000000000000272

Peters-Veluthamaningal, C., Van Der Windt, D. A. W. M., Winters, J. C., & Meyboom-de Jong, B. (2009). Corticosteroid injection for de Quervain's tenosynovitis. *Cochrane Database of Systematic Reviews*, (3). https://doi.org/10.1002/14651858.CD005616.pub2

Walker-Bone, K., Palmer, K. T., Reading, I., Coggon, D., & Cooper, C. (2004). Prevalence and impact of musculoskeletal disorders of the upper limb in the general population. *Arthritis Care & Research*, *51*(4), 642–651. https://doi.org/10.1002/art.20535

Research question template

The research question is:

Do standardised screening tools improve early diagnosis of osteoarthritis (OA)?

Please tick one box for each of the following categories:

Category	Content relevant to this research question	Your rating of this category
Extent to which the question is important to patients and other health decision-makers	Patients, researchers, and clinicians have all shown to prioritise self-help and education in the management of OA, with patients also showing preference for conservative care. If early diagnosis can be achieved through screening then management is likely to slow progression and reduce impact and burden (Schofield, Shrestha, & Cunich, 2016) and may also alleviate patient perception that OA is inevitable and untreatable (Alami et al., 2011). Accurate diagnosis of OA is important to patients, especially younger ones, who report frustrations with a long diagnostic process (Gignac et al., 2006). Conversely, early diagnosis doesn't appear to be important to some clinicians who perceive OA diagnosis to be trivial and not crucial for treatment (Alami et al., 2011).	☐ Not shown to be important to either patients or decision-makers<br>☐ Shown to be important to health decision-makers but not patients<br>☐ Shown to be important to patients but not decision-makers<br>☐ Shown to be important to both patients and decision-makers
That it addresses an area of high patient burden	Although non-lethal, osteoarthritis can significantly affect the lives of individuals, with pain and stiffness limiting activities of daily living leading to depression and impacting quality of sleep (Bijlsma, Berenbaum, & Lafeber, 2011). These symptoms can limit ability to work and, in many cases, require help from carers.<br><br>In Australia, osteoarthritis accounts for approximately 2% of the burden of disease, resulting in 83,405 DALYs in 2011. Similarly, the burden of disease is increasing in New Zealand and OA accounted for approximately 1% (10,000 DALY) in 2013.	☐ Mild symptoms and little or no associated disability<br>☐ Moderate symptoms and some disability<br>☐ Significantly disabling, associated with mortality risk or no effective treatments available
That it addresses an area of high social burden	Osteoarthritis of the hip and knee was ranked 11th highest global contributor to disability, with a prevalence of 3.8% and 0.85% for knee and hip respectively (Cross et al., 2014). In Australia the age-adjusted prevalence of diagnosed osteoarthritis is 8.1% (Australian Institute of Health and Welfare, 2018) and in New Zealand it is 7.2% (Ministry of Health, 2017). With substantially higher incidences occurring in women and those aged over 65, these figures are set to increase due to the rising levels of obesity and an aging population.	☐ Condition is rare<br>☐ Condition is somewhat common<br>☐ Condition is common<br>☐ Condition is highly prevalent

Potential reduction in patient and/or social burden due to (clinical or implementation) intervention	While no cure exists, early detection and intervention can slow progressions, reduce disability, reduce absenteeism, and greatly improve the way of life. A scalable model of care would likely improve outcomes through targeted weight loss, physical activity, allied care, medication and management in order to delay or reduce surgical intervention (Baldwin, Briggs, Bagg, & Larmer, 2017). The two major risk factors, obesity and joint injury, are both modifiable and early detection may greatly impede disease progression across an entire population.	☐ Symptomatic treatment only and small potential effect size<br>☐ Symptomatic treatment and large potential effect size<br>☐ Potential for intervention to treat both symptoms and underlying disease pathology<br>☐ Potential for cure or fundamental alteration of disease course
Potential scalability and uptake or intervention	Diagnosis of OA normally occurs upon presentation once the disease has progressed enough to impact quality of life. Development of a standardised screening tools may assist in early detection. Simple questionnaires administered by non-medical staff have shown promising results (Quintana et al., 2007; Roux et al., 2008) and could be developed in to a screening tool with relatively little training or expenditure.	☐ Low potential for scalability and uptake<br>☐ High potential for uptake but low scalability<br>☐ High potential for scalability but low potential for uptake<br>☐ High potential for both scalability and uptake
Extent to which the question addresses health equity	Education level is associated with prevalence of hip and knee OA, as well as likelihood of progression, need for total hip replacement, and unmet need for arthroplasty (Luong, Cleveland, Nyrop, & Callahan, 2012).<br><br>In Australia, a higher prevalence of OA in areas of most deprivation or lowest socioeconomic status (Australian Institute of Health and Welfare, 2018) suggests those affected are more likely to have manual jobs and may be most impacted by loss of work or lack of access to health.	☐ No attempt to address health equity<br>☐ Discussed using Progress Plus items (O'Neill et al., 2014) but intervention not relevant or appropriate<br>☐ Intervention shown to have some potential application to improving health equity issues<br>☐ The intervention is explicitly designed to improve health equity issues

Final question:

In your opinion, do you think this research question is important enough for ANZMUSC to endorse it (assuming all other endorsement criteria are met)?

☐ Yes

☐ No

References

Alami, S., Boutron, I., Desjeux, D., Hirschhorn, M., Meric, G., Rannou, F., & Poiraudeau, S. (2011). Patients' and practitioners' views of knee osteoarthritis and its management: A qualitative interview study. *PLoS ONE*, *6*(5), e19634. https://doi.org/10.1371/journal.pone.0019634

Australian Institute of Health and Welfare. (2018). Osteoarthritis snapshot. Retrieved September 11, 2018, from https://www.aihw.gov.au/reports/chronic-musculoskeletal-conditions/osteoarthritis/data

Baldwin, J., Briggs, A., Bagg, W., & Larmer, P. (2017). An osteoarthritis model of care should be a national priority for New Zealand. *NZMJ*, *130*, 1467. Retrieved from www.nzma.org.nz/journal

Bijlsma, J. W. J., Berenbaum, F., & Lafeber, F. P. J. G. (2011). Osteoarthritis: An update with relevance for clinical practice. *The Lancet*, *377*(9783), 2115–2126. https://doi.org/10.1016/S0140-6736(11)60243-2

Cross, M., Smith, E., Hoy, D., Nolte, S., Ackerman, I., Fransen, M., … March, L. (2014). The global burden of hip and knee osteoarthritis: Estimates from the Global Burden of Disease 2010 study. *Annals of the Rheumatic Diseases*, *73*(7), 1323–1330. https://doi.org/10.1136/annrheumdis-2013-204763

Gignac, M. A. M., Davis, A. M., Hawker, G., Wright, J. G., Mahomed, N., Fortin, P. R., & Badley, E. M. (2006). "What do you expect? You're just getting older": A comparison of perceived osteoarthritis-related and aging-related health experiences in middle- and older-age adults. *Arthritis Care and Research*, *55*(6), 905–912. https://doi.org/10.1002/art.22338

Luong, M. L. N., Cleveland, R. J., Nyrop, K. A., & Callahan, L. F. (2012). Social determinants and osteoarthritis outcomes. *Aging Health*, *8*(4), 413–437. https://doi.org/10.2217/ahe.12.43

Ministry of Health. (2017). *Annual Data Explorer 2016/17: New Zealand Health Survey [Data File]*. Retrieved from https://minhealthnz.shinyapps.io/nz-health-survey-2016-17-annual-update

Quintana, J. M., Arostegui, I., Escobar, A., Lafuente, I., Arenaza, J. C., Garcia, I., & Aguirre, U. (2007). Validation of a screening questionnaire for hip and knee osteoarthritis in old people. https://doi.org/10.1186/1471-2474-8-84

Roux, C. H., Saraux, A., Mazieres, B., Pouchot, J., Morvan, J., Fautrel, B., … Euller-Ziegler, L. (2008). Screening for hip and knee osteoarthritis in the general population: Predictive value of a questionnaire and prevalence estimates. *Annals of the Rheumatic Diseases*, *67*(10), 1406–1411. https://doi.org/10.1136/ard.2007.075952

Schofield, D., Shrestha, R., & Cunich, M. (2016). *Counting the cost Part 2 Economic costs: Current and future burden of arthritis*. Sydney. Retrieved from https://www.arthritistas.org.au/wp-content/uploads/2016/05/Final-Counting-the-Costs_Part2_Economic-Costs-MAY2016.pdf

Research question template

The research question is:

Is pregabalin effective for acute and chronic sciatica?

Please tick one box for each of the following categories:

Category	Content relevant to this research question	Your rating of this category
Extent to which the question is important to patients and other health decision-makers	Effective pain management is a high priority for patients experiencing sciatica as opioids have little effect on treating neurogenic pain. Clinicians too are seeking for further research on effective pharmacological interventions.	☐ Not shown to be important to either patients or decision-makers<br>☐ Shown to be important to health decision-makers but not patients<br>☐ Shown to be important to patients but not decision-makers<br>☐ Shown to be important to both patients and decision-makers
That it addresses an area of high patient burden	Sciatica is a description of symptoms arising from nerve root irritation, primarily caused by disc herniation. Patients experience low back pain and pain that extends beyond the knee. Neurological symptoms are often present such as paraesthesia and weakness. In the majority of cases, patients recover within three months with surgical intervention or with conservative care, although in 30% of cases the patient will experience pain longer than 12 months (Koes, van Tulder, & Peul, 2007).	☐ Mild symptoms and little or no associated disability<br>☐ Moderate symptoms and some disability<br>☐ Significantly disabling, associated with mortality risk or no effective treatments available
That it addresses an area of high social burden	Prevalence data for sciatica symptoms is sparse due to poor consensus on definitions and testing. Worldwide period prevalence varies between 1.6% and 43% (Konstantinou & Dunn, 2008) with New Zealand study measuring period prevalence by examining low back pain with referred pain in the buttock (9.5%), in to the thigh (9%), and beyond the knee (4.9%) (Laslett, Crothers, Beattie, Cregten, & Moses, 1991).	☐ Condition is rare<br>☐ Condition is somewhat common<br>☐ Condition is common<br>☐ Condition is highly prevalent

Potential reduction in patient and/or social burden due to (clinical or implementation) intervention	Sciatica is the symptomatic manifestation of a variety of low back conditions creating lumbosacral neurogenic pain such as disc herniation, spondylosis, or inflammatory conditions (Tarulli & Raynor, 2007). Pregabalin appears to have some benefit in managing symptoms in patients with radiculopathy (Saldaña, Navarro, Pérez, Masramón, & Rejas, 2010), however its analgesic mechanism of action is purely symptomatic and is unlikely to resolve any underlying pathology.	☐ Symptomatic treatment only and small potential effect size<br>☐ Symptomatic treatment and large potential effect size<br>☐ Potential for intervention to treat both symptoms and underlying disease pathology<br>☐ Potential for cure or fundamental alteration of disease course
Potential scalability and uptake or intervention	Pregabalin is readily available as a funded medicine in Australia and New Zealand for the management of treatment resistant neuropathic pain and for the control of seizures (NPS MedicineWise, 2013). Although there are minimal side effects, some evidence of abuse exists when taken alongside recreational drugs and prescribers should be aware of potential for misuse (Ponton, 2018).	☐ Low potential for scalability and uptake<br>☐ High potential for uptake but low scalability<br>☐ High potential for scalability but low potential for uptake<br>☐ High potential for both scalability and uptake
Extent to which the question addresses health equity	Data pertaining to the health equity of patients with sciatica is sparse. As a subset category of low back pain, however, it is likely that higher levels of disability will be found in low-income countries, areas of high deprivation, and in people working in jobs with high levels of manual labour (Clark & Horton, 2018).	☐ No attempt to address health equity<br>☐ Discussed using Progress Plus items (O'Neill et al., 2014) but intervention not relevant or appropriate<br>☐ Intervention shown to have some potential application to improving health equity issues<br>☐ The intervention is explicitly designed to improve health equity issues

Final question:

In your opinion, do you think this research question is important enough for ANZMUSC to endorse it (assuming all other endorsement criteria are met)?

☐ Yes

☐ No

References

Clark, S., & Horton, R. (2018). Low back pain: a major global challenge. *The Lancet*, *391*(10137), 2302. https://doi.org/10.1016/S0140-6736(18)30725-6

Koes, B. W., van Tulder, M. W., & Peul, W. C. (2007). Diagnosis and treatment of sciatica. *BMJ*, *334*(7607), 1313–1317. https://doi.org/10.1136/bmj.39223.428495.BE

Konstantinou, K., & Dunn, K. M. (2008). Sciatica: Review of epidemiological studies and prevalence estimates. *Spine*, *33*(22), 2464–2472. https://doi.org/10.1097/BRS.0b013e318183a4a2

Laslett, M., Crothers, C., Beattie, P., Cregten, L., & Moses, A. (1991). The frequency and incidence of low back pain/sciatica in an urban population. *New Zealand Medical Journal*, *104*(921), 424–426. Retrieved from http://aut.researchgateway.ac.nz/handle/10292/1373

NPS MedicineWise. (2013). Pregabalin (Lyrica) for neuropathic pain. Retrieved November 27, 2018, from https://www.nps.org.au/radar/articles/pregabalin-lyrica-for-neuropathic-pain#safety-issues

Ponton, R. (2018). Pregabalin misuse: preventing potential problems in New Zealand. *New Zealand Medical Journal*, *131*(1478). Retrieved from https://www.nzma.org.nz/journal/read-the-journal/all-issues/2010-2019/2018/vol-131-no-1478-13-july-2018/7621

Saldaña, M. T., Navarro, A., Pérez, C., Masramón, X., & Rejas, J. (2010). Patient-reported-outcomes in subjects with painful lumbar or cervical radiculopathy treated with pregabalin: Evidence from medical practice in primary care settings. *Rheumatology International*, *30*(8), 1005–1015. https://doi.org/10.1007/s00296-009-1086-1

Tarulli, A. W., & Raynor, E. M. (2007). Lumbosacral Radiculopathy. *Neurologic Clinics*, *25*(2), 387–405. https://doi.org/10.1016/j.ncl.2007.01.008

Research question template

The research question is:

Is pooled faecal microbiota transplantation (FMT) as effective as adalimumab in patients with axial spondyloarthritis (axSpA)?

Please tick one box for each of the following categories:

Category	Content relevant to this research question	Your rating of this category
Extent to which the question is important to patients and other health decision-makers	In research examining patient perceptions to FMT in ulcerative colitis, there was overwhelming support and interest for its use. This was reportedly due to its 'natural' mechanism of action and frustration of a lack of cure for the patient's disease, particularly in more severe cases (Kahn et al., 2013). Clinicians and researchers too, are interested in the continued development of FMT in the treatment of disease although feel hampered by policy restrictions that slows research, such as the classification of FMT as a medicine and not human tissue by the FDA (Sofi, Georgescu, Sodeman, & Nawras, 2013).	☐ Not shown to be important to either patients or decision-makers<br>☐ Shown to be important to health decision-makers but not patients<br>☐ Shown to be important to patients but not decision-makers<br>☐ Shown to be important to both patients and decision-makers
That it addresses an area of high patient burden	Ankylosing spondylitis, the fully progressed form of axSpa, is a debilitating and painful disease with quality of life scores (SF36) comparable to patients with chronic heart failure. As well as severe spinal rigidity and pain, reduced bone density in 50% of cases leads to a risk of substantial risk of spinal fractures and patients are 11 times more likely to experience spinal cord injury. Comorbidities include uveitis, inflammatory bowel disease, and psoriasis. Patients with severe spinal changes often experience breathing difficulty and myocardial infarction. Withdrawal from the workforce is as high as 36% of patients, and those still working regularly take long periods of sick leave. (Boonen & Linden, 2006)	☐ Mild symptoms and little or no associated disability<br>☐ Moderate symptoms and some disability<br>☐ Significantly disabling, associated with mortality risk or no effective treatments available
That it addresses an area of high social burden	Globally, axSpA is a relatively common condition with a prevalence of 0.36-0.7%. It occurs more frequently in males aged 40 years or older. Prevalence is higher in North American and European populations and is markedly higher in indigenous arctic communities (Stolwijk, van Onna, Boonen, & van Tubergen, 2016).<br><br>In Australia and New Zealand there is limited data as to the prevalence and estimates are made of roughly 0.5% (1 in 200 people). In Maori or Aboriginal peoples, it is highly likely that rheumatic diseases were not present until European colonialization and (Roberts-Thomson & Roberts-Thomson, 1999; Roberts et al., 2013) and therefore a lower prevalence is expected.	☐ Condition is rare<br>☐ Condition is somewhat common<br>☐ Condition is common<br>☐ Condition is highly prevalent

Potential reduction in patient and/or social burden due to (clinical or implementation) intervention	Early detection and modification of gut microbiota in early non-radiographic stages of axSpA may prevent progression of the disease in to ankylosing spondylitis. While treatment using adalimumab is shown to be effective in reducing inflammation of the axial skeleton, FMT has the potential to cure the underlying pathological process.<br><br>As seen in the remission of crohns disease following FMT trials, the use of carefully selected microbial species has the potential to ameliorate axSpA triggered by HLA-B27 and animal models have shown good success in this process (Stoll, 2014). Restoration of microbial structure similar to that found in donors occurs as quickly as 24 hours following FMT (Weingarden et al., 2015) which suggests the potential to quickly reverse the auto-inflammatory processes.	☐ Symptomatic treatment only and small potential effect size<br>☐ Symptomatic treatment and large potential effect size<br>☐ Potential for intervention to treat both symptoms and underlying disease pathology<br>☐ Potential for cure or fundamental alteration of disease course
Potential scalability and uptake or intervention	Poor understanding of the complexity of gut microbiota alongside the inability grow many in culture creates dependence on donors. Currently, screening of donors is difficult and the potential for recipients to develop donor traits such as obesity or even autoimmune diseases is poorly understood (Khoruts & Sadowsky, 2016).<br><br>Beyond donor selection, the collection, preparation, storage and administration are uncomplicated processes (Cammarota et al., 2017) and once our understanding has progressed the use of FMT has the potential to become a simple and widespread therapy for gut-microbiota related inflammatory disorders.	☐ Low potential for scalability and uptake<br>☐ High potential for uptake but low scalability<br>☐ High potential for scalability but low potential for uptake<br>☐ High potential for both scalability and uptake
Extent to which the question addresses health equity	Ankylosing spondylitis prevalence is three times higher in Canadian and Alaskan First Nations people (Barnabe et al., 2017). Similar to New Zealand Maori and indigenous Australians suffering from rheumatic conditions, these peoples are more likely to be hospitalised for their symptoms but less likely to see a specialist.<br><br>The disease is also more likely to occur in rural populations (Stolwijk, Boonen, van Tubergen, & Reveille, 2012) which may increase the burden of disease due to a greater withdrawal from work seen in those with manual jobs (Boonen & Linden, 2006) and limited access to health services.	☐ No attempt to address health equity<br>☐ Discussed using Progress Plus items (O'Neill et al., 2014) but intervention not relevant or appropriate<br>☐ Intervention shown to have some potential application to improving health equity issues<br>☐ The intervention is explicitly designed to improve health equity issues

Final question:

In your opinion, do you think this research question is important enough for ANZMUSC to endorse it (assuming all other endorsement criteria are met)?

☐ Yes

☐ No

References

Barnabe, C., Jones, C. A., Bernatsky, S., Peschken, C. A., Voaklander, D., Homik, J., … Hemmelgarn, B. (2017). Inflammatory Arthritis Prevalence and Health Services Use in the First Nations and Non–First Nations Populations of Alberta, Canada. *Arthritis Care and Research*, *69*(4), 467–474. https://doi.org/10.1002/acr.22959

Boonen, A., & Linden, S. M. Van Der. (2006). The Burden of Ankylosing Spondylitis LIFE. *Journal of Rheumatology*, *78*, 4–11. Retrieved from http://www.jrheum.org/content/78/4.long

Cammarota, G., Ianiro, G., Tilg, H., Rajilić-Stojanović, M., Kump, P., Satokari, R., … Gasbarrini, A. (2017). European consensus conference on faecal microbiota transplantation in clinical practice. In *Gut* (Vol. 66, pp. 569–580). https://doi.org/10.1136/gutjnl-2016-313017

Kahn, S. A., Vachon, A., Rodriquez, D., Goeppinger, S. R., Surma, B., Marks, J., & Rubin, D. T. (2013). Patient perceptions of fecal microbiota transplantation for ulcerative colitis. *Inflammatory Bowel Diseases*, *19*(7), 1506–1513. https://doi.org/10.1097/MIB.0b013e318281f520

Khoruts, A., & Sadowsky, M. J. (2016). Understanding the mechanisms of faecal microbiota transplantation. *Nature Reviews Gastroenterology and Hepatology*, *13*(9), 508–516. https://doi.org/10.1038/nrgastro.2016.98

Roberts-Thomson, R. A., & Roberts-Thomson, P. J. (1999). Rheumatic disease and the Australian Aborigine. *Annals of the Rheumatic Diseases*, *58*(5), 266–270. https://doi.org/10.1136/ard.58.5.266

Roberts, R. L., Wallace, M. C., Jones, G. T., van Rij, A. M., Merriman, T. R., Harrison, A., … Stebbings, S. M. (2013). Prevalence of HLA-B27 in the New Zealand population: Effect of age and ethnicity. *Arthritis Research and Therapy*, *15*(5), R158. https://doi.org/10.1186/ar4341

Sofi, A. A., Georgescu, C., Sodeman, T., & Nawras, A. (2013). Physician outlook toward fecal microbiota transplantation in the treatment of Clostridium difficile infection. *American Journal of Gastroenterology*, *108*(10), 1661–1662. https://doi.org/10.1038/ajg.2013.207

Stoll, M. L. (2014). Gut microbes, immunity, and spondyloarthritis. *Clinical Immunology*, *159*(2), 134–142. https://doi.org/10.1016/j.clim.2015.05.001

Stolwijk, C., Boonen, A., van Tubergen, A., & Reveille, J. D. (2012). Epidemiology of Spondyloarthritis. *Rheumatic Disease Clinics of North America*, *38*(3), 441–476. https://doi.org/10.1016/j.rdc.2012.09.003

Stolwijk, C., van Onna, M., Boonen, A., & van Tubergen, A. (2016). Global Prevalence of Spondyloarthritis: A Systematic Review and Meta-Regression Analysis. *Arthritis Care & Research*, *68*(9), 1320–1331. https://doi.org/10.1002/acr.22831

Weingarden, A., González, A., Vázquez-Baeza, Y., Weiss, S., Humphry, G., Berg-Lyons, D., … Sadowsky, M. J. (2015). Dynamic changes in short- and long-term bacterial composition following fecal microbiota transplantation for recurrent Clostridium difficile infection. *Microbiome*, *3*(1), 10. https://doi.org/10.1186/s40168-015-0070-0

Research question template

The research question is:

In patients with symptomatic degenerative rotator cuff tears, does surgery provide superior pain and function improvement compared to placebo surgery?

Please tick one box for each of the following categories:

Category	Content relevant to this research question	Your rating of this category
Extent to which the question is important to patients and other health decision-makers	Many patients perceive surgery to be the last resort of rotator cuff repair if conservative therapies fail, but feel pressured by clinicians to skip physical therapies and rehabilitative therapies. Strong evidence showing the efficacy of surgery over placebo may help to alleviate patient concerns over surgical intervention.	☐ Not shown to be important to either patients or decision-makers<br>☐ Shown to be important to health decision-makers but not patients<br>☐ Shown to be important to patients but not decision-makers<br>☐ Shown to be important to both patients and decision-makers
That it addresses an area of high patient burden	Rotator cuff tears can be moderately disabling, resulting in significant pain that can last for over a year. Pain associated with overhead movements can limit young person's ability to work or play sports and limit activities of daily living in older patients. Rotator cuff tears are also associated with a decreased quality of mental health (Piitulainen, Ylinen, Kautiainen, & Häkkinen, 2012).	☐ Mild symptoms and little or no associated disability<br>☐ Moderate symptoms and some disability<br>☐ Significantly disabling, associated with mortality risk or no effective treatments available
That it addresses an area of high social burden	Prevalence of rotator cuff tears increases with age, from 9.2% in those 20 years and younger to 62% in those aged 80 years or more, suggesting that degeneration is a natural process of aging (Teunis, Lubberts, Reilly, & Ring, 2014). Tears are the biggest cause for shoulder pain and in the year ending Jun 2018, The Accident Compensation Corporation of New Zealand received 125,684 new claims for soft tissue injuries to the shoulder costing NZD $243M (Accident Compensaion Corporation, 2018)	☐ Condition is rare<br>☐ Condition is somewhat common<br>☐ Condition is common<br>☐ Condition is highly prevalent

Potential reduction in patient and/or social burden due to (clinical or implementation) intervention	In a review of surgery vs placebo surgery studies it was found that patients symptoms improved regardless of type of surgery, only half of surgeries were found to be superior to placebo surgery, and in most cases the size of the effect was small (Wartolowska et al., 2014). Despite a steady increase in shoulder surgery for rotator cuff disorders within Australia (Thorpe, Hurworth, O'Sullivan, Mitchell, & Smith, 2016), a distinct lack of evidence exists suggesting that surgery provides any greater cure when compared to conservative therapies (Coghlan, Buchbinder, Green, Johnston, & Bell, 2008).	☐ Symptomatic treatment only and small potential effect size<br>☐ Symptomatic treatment and large potential effect size<br>☐ Potential for intervention to treat both symptoms and underlying disease pathology<br>☐ Potential for cure or fundamental alteration of disease course
Potential scalability and uptake or intervention	Although the shoulder surgery is a complex procedure with a steep learning curve (Groh & Groh, 2014), the number of arthroscopic procedures in the Australia has increased by 103% for arthroscopic subacromial decompression and 68% for arthroscopic reconstruction between 2001 and 2013 (Thorpe et al., 2016). This increase may suggest that, if found to be effective, the operation could be scaled up throughout Australia and New Zealand with extended training for new and existing surgeons.	☐ Low potential for scalability and uptake<br>☐ High potential for uptake but low scalability<br>☐ High potential for scalability but low potential for uptake<br>☐ High potential for both scalability and uptake
Extent to which the question addresses health equity	Few or no studies have examined the relationship of rotator cuff tears to health equity items. Beyond increased risk for tears associated with heavy labour type jobs (Yamamoto et al., 2010) and smoking (Baumgarten et al., 2010), there does not appear to be any increased risk related with ethnicity, or with co-morbidities commonly found in areas of higher deprivation (Fehringer, Sun, VanOeveren, Keller, & Matsen, 2008).	☐ No attempt to address health equity<br>☐ Discussed using Progress Plus items (O'Neill et al., 2014) but intervention not relevant or appropriate<br>☐ Intervention shown to have some potential application to improving health equity issues<br>☐ The intervention is explicitly designed to improve health equity issues

Final question:

In your opinion, do you think this research question is important enough for ANZMUSC to endorse it (assuming all other endorsement criteria are met)?

☐ Yes

☐ No

References

Accident Compensaion Corporation. (2018). Statistics on our claims. Retrieved November 5, 2018, from https://www.acc.co.nz/about-us/statistics/

Baumgarten, K. M., Gerlach, D., Galatz, L. M., Teefey, S. A., Middleton, W. D., Ditsios, K., & Yamaguchi, K. (2010). Cigarette smoking increases the risk for rotator cuff tears. *Clinical Orthopaedics and Related Research*, *468*(6), 1534–1541. https://doi.org/10.1007/s11999-009-0781-2

Coghlan, J. A., Buchbinder, R., Green, S., Johnston, R. V, & Bell, S. N. (2008). Surgery for rotator cuff disease. *Cochrane Database of Systematic Reviews*, (1). https://doi.org/10.1002/14651858.CD005619.pub2

Fehringer, E. V., Sun, J., VanOeveren, L. S., Keller, B. K., & Matsen, F. A. (2008). Full-thickness rotator cuff tear prevalence and correlation with function and co-morbidities in patients sixty-five years and older. *Journal of Shoulder and Elbow Surgery*, *17*(6), 881–885. https://doi.org/10.1016/j.jse.2008.05.039

Groh, G. I., & Groh, G. M. (2014). Complications rates, reoperation rates, and the learning curve in reverse shoulder arthroplasty. *Journal of Shoulder and Elbow Surgery*, *23*(3), 388–394. https://doi.org/10.1016/J.JSE.2013.06.002

Piitulainen, K., Ylinen, J., Kautiainen, H., & Häkkinen, A. (2012). The relationship between functional disability and health-related quality of life in patients with a rotator cuff tear. *Disability and Rehabilitation*, *34*(24), 2071–2075. https://doi.org/10.3109/09638288.2012.670363

Teunis, T., Lubberts, B., Reilly, B. T., & Ring, D. (2014). A systematic review and pooled analysis of the prevalence of rotator cuff disease with increasing age. *Journal of Shoulder and Elbow Surgery*, *23*(12), 1913–1921. https://doi.org/10.1016/j.jse.2014.08.001

Thorpe, A., Hurworth, M., O'Sullivan, P., Mitchell, T., & Smith, A. (2016). Rising trends in surgery for rotator cuff disease in Western Australia. *ANZ Journal of Surgery*, *86*(10), 801–804. https://doi.org/10.1111/ans.13691

Wartolowska, K., Judge, A., Hopewell, S., Collins, G. S., Dean, B. J. F., Rombach, I., … Carr, A. J. (2014). Use of placebo controls in the evaluation of surgery: Systematic review. *BMJ (Online)*, *348*, g3253. https://doi.org/10.1136/bmj.g3253

Yamamoto, A., Takagishi, K., Osawa, T., Yanagawa, T., Nakajima, D., Shitara, H., & Kobayashi, T. (2010). Prevalence and risk factors of a rotator cuff tear in the general population. *Journal of Shoulder and Elbow Surgery*, *19*(1), 116–120. https://doi.org/10.1016/j.jse.2009.04.006

Research question template

The research question is:

What (health service) pre-operative, intra-operative, and post-operative factors can be modified to influence outcome following hip and knee replacement?

Please tick one box for each of the following categories:

Category	Content relevant to this research question	Your rating of this category
Extent to which the question is important to patients and other health decision-makers	Many osteoarthritis patients prioritise joint replacement and education as their primary treatment priorities, whereas much of the research is focused on pharmacological interventions and pain management (Tallon, Chard, & Dieppe, 2000). Furthering the research agenda beyond pharmaceuticals will allow patients undergoing their preferred treatment to receive the the best education, management and recovery outcomes. This is also reflected in research examining clinicians priorities, where many are looking to make improvements in total knee and hip joint replacements and want more research on pain management, patient education, and rehabilitation (Westby & Backman, 2010).	☐ Not shown to be important to either patients or decision-makers<br>☐ Shown to be important to health decision-makers but not patients<br>☐ Shown to be important to patients but not decision-makers<br>☐ Shown to be important to both patients and decision-makers
That it addresses an area of high patient burden	Outcome measures following hip and knee arthroplasty pain levels, function, and emotional improvements. Pain can remain in as many as 30% of patients postoperatively and can be impacted by socioeconomic deprivation, patient psychological factors such as anxiety or depression, or back pain (Lungu, Vendittoli, & Desmeules, 2016; Sullivan et al., 2009).	☐ Mild symptoms and little or no associated disability<br>☐ Moderate symptoms and some disability<br>☐ Significantly disabling, associated with mortality risk or no effective treatments available
That it addresses an area of high social burden	In Australia there were 47,972 hip replacements and 63,854 knee replacements in 2018 which represents a 77.6% and 123.5% increase respectively since 2003 (Australian Orthopaedic Association National Joint Replacement Registry, 2018). While the procedure is very effective, negative outcomes are common including 5% of patients experiencing surgical site infection, 5% experiencing venous thromboembolism, and 5% experiencing post-surgical delirium in a large Melbourne cohort (Peel et al., 2015)	☐ Condition is rare<br>☐ Condition is somewhat common<br>☐ Condition is common<br>☐ Condition is highly prevalent

Potential reduction in patient and/or social burden due to (clinical or implementation) intervention	Effective arthroplasty has the ability to remove damaged and disease bone and tissue to effectively overcome disease such as osteoarthritis where other modalities are unable to. However this intervention does not always resolve pain and functional outcomes, due to influencing biopsychosocial factors. Understanding how to manage and mitigate any negative outcomes may help to improve outcomes and provide permanent relief for patients.	☐ Symptomatic treatment only and small potential effect size<br>☐ Symptomatic treatment and large potential effect size<br>☐ Potential for intervention to treat both symptoms and underlying disease pathology<br>☐ Potential for cure or fundamental alteration of disease course
Potential scalability and uptake or intervention	Depending on factors found to produce favourable outcomes, implementation will range in complexity and scalability and may require substantial changes in best practices. Outcomes can be influenced by disease progression (Fortin et al., 1999), surgeon skill and busyness (Heck, Robinson, Partridge, Lubitz, & Freund, 1998), and patient preoperative mental state (Judge et al., 2012). Incorporating changes may require changes to social services, rehabilitative goals, surgical procedures, hardware, and technology.	☐ Low potential for scalability and uptake<br>☐ High potential for uptake but low scalability<br>☐ High potential for scalability but low potential for uptake<br>☐ High potential for both scalability and uptake
Extent to which the question addresses health equity	There is inequity in access to hip and knee replacements by age, deprivation, and ethnicity even after adjusting for hospital distance (Judge & Welton, 2007) and those living in the highest deprivation experienced the poorer outcomes (Judge et al., 2012; Lungu et al., 2016). New Zealand Maori were found to have worse pre-operative functional scores, worse post-operative functional scores, and smaller improvements following knee and hip replacements (Singleton, Buddicom, Vane, & Poutawera, 2013).	☐ No attempt to address health equity<br>☐ Discussed using Progress Plus items (O'Neill et al., 2014) but intervention not relevant or appropriate<br>☐ Intervention shown to have some potential application to improving health equity issues<br>☐ The intervention is explicitly designed to improve health equity issues

Final question:

In your opinion, do you think this research question is important enough for ANZMUSC to endorse it (assuming all other endorsement criteria are met)?

☐ Yes

☐ No

References

Australian Orthopaedic Association National Joint Replacement Registry. (2018). *Hip, Knee & Shoulder Arthroplasty: 2018 Annual Report*. Adelaide. Retrieved from www.aoa.org.au

Fortin, P. R., Clarke, A. E., Joseph, L., Liang, M. H., Tanzer, M., Ferland, D., … Katz, J. N. (1999). Outcomes of total hip and knee replacement: Preoperative functional status predicts outcomes at six months after surgery. *Arthritis and Rheumatism*, *42*(8), 1722–1728. https://doi.org/10.1002/1529-0131(199908)42:8<1722::AID-ANR22>3.0.CO;2-R

Heck, D. A., Robinson, R. L., Partridge, C. M., Lubitz, R. M., & Freund, D. A. (1998). Patient Outcomes After Knee Replacement. *Clinical Orthopaedics and Related Research*, *356*(356), 93–110. Retrieved from http://journals.lww.com/corr/Abstract/1998/11000/Patient_Outcomes_After_Knee_Replacement.15.aspx

Judge, A., Arden, N. K., Cooper, C., Kassim javaid, M., Carr, A. J., Field, R. E., & Dieppe, P. A. (2012). Predictors of outcomes of total knee replacement surgery. *Rheumatology (United Kingdom)*, *51*(10), 1804–1813. https://doi.org/10.1093/rheumatology/kes075

Judge, A., & Welton, N. J. (2007). Equity in access to total joint replacement of the hip and knee in England: cross sectional study. https://doi.org/10.1136/bmj.c4092

Lungu, E., Vendittoli, P.-A., & Desmeules, F. (2016). Preoperative Determinants of Patient-reported Pain and Physical Function Levels Following Total Knee Arthroplasty: A Systematic Review. *The Open Orthopaedics Journal*, *10*, 213–231. https://doi.org/10.2174/1874325001610010213

Peel, T. N., Cheng, A. C., Liew, D., Buising, K. L., Lisik, J., Carroll, K. A., … Dowsey, M. M. (2015). Direct hospital cost determinants following hip and knee arthroplasty. *Arthritis Care and Research*, *67*(6), 782–790. https://doi.org/10.1002/acr.22523

Singleton, N., Buddicom, E., Vane, A., & Poutawera, V. (2013). Are there differences between Maori and non-Maori patients undergoing primary total hip and knee arthroplasty surgery in New Zealand? A registry-based cohort study. *The New Zealand Medical Journal*, *126*(1379), 23–30. Retrieved from https://www.nzma.org.nz/journal/read-the-journal/all-issues/2010-2019/2013/vol-126-no-1379/article-singleton

Sullivan, M., Tanzer, M., Stanish, W., Fallaha, M., Keefe, F. J., Simmonds, M., & Dunbar, M. (2009). Psychological determinants of problematic outcomes following Total Knee Arthroplasty. *Pain*, *143*(1–2), 123–129. https://doi.org/10.1016/J.PAIN.2009.02.011

Tallon, D., Chard, J., & Dieppe, P. (2000). Relation between agendas of the research community and the research consumer. *The Lancet*, *355*. https://doi.org/10.1016/S0140-6736(00)02351-5

Westby, M. D., & Backman, C. L. (2010). Patient and health professional views on rehabilitation practices and outcomes following total hip and knee arthroplasty for osteoarthritis: A focus group study. *BMC Health Services Research*, *10*(1), 119. https://doi.org/10.1186/1472-6963-10-119

Research question template

The research question is:

Are workplace interventions efficacious for treating work disability due to low back pain (LBP)?

Please tick one box for each of the following categories:

Category	Content relevant to this research question	Your rating of this category
Extent to which the question is important to patients and other health decision-makers	Patients undergoing treatment for LBP regularly report dissatisfaction with medication, a preference for conservative care, and poor adherence to physical activity. While little evidence exists specifically in the context of the workplace, major barrier to exercise for LBP include a lack of time due to professional requirements and a lack of colleague support (Mathy, Cedraschi, Broonen, Azzi, & Henrotin, 2015). Normalising workplace interventions such as exercises may address these occupational barriers and encourage better self-care through structured programmes for the management of LBP.<br><br>Primary care providers dealing with LBP report that occupational management is a priority. A 2013 survey of 145 researchers and clinicians showed that finding effective treatments, effective self-care strategies, effective workplace interventions, and facilitating return to work were ranked highly for future research (Costa et al., 2013). Policy makers in New Zealand are also concerned over overspending on ineffective treatment methods such as spinal surgery and have prioritised the review of the model of care for back pain (National Health Committee, 2015), with allied providers and researchers calling for the similar reviews in Australia (Briggs & Buchbinder, 2009).	☐ Not shown to be important to either patients or decision-makers<br>☐ Shown to be important to health decision-makers but not patients<br>☐ Shown to be important to patients but not decision-makers<br>☐ Shown to be important to both patients and decision-makers
That it addresses an area of high patient burden	LBP symptoms include high levels of pain which can be long lasting and heavily impact quality of life. In acute patients the median duration of pain is 15 days and in chronic patients the median duration of pain is 128 days. Associated physiological harm can lead to depression and societal withdrawal. There is poor evidence regarding the mortality risks associated with LBP.<br>(D. Hoy, Brooks, Blyth, & Buchbinder, 2010)<br><br>In New Zealand, back disorders were the first and second causes of health loss in 2013 in females (9.7% total DALY) and males (8.2% total DALY) respectively (Ministry of Health, 2016). Self-reported absenteeism in NZ is 9% of adults per year, with 28% of adults reporting reduced activities (Widanarko et al., 2012). In Australia, back disorders accounted for 4% of total DALYs in 2011 (Australian Institute of Health and Welfare, 2017) with similar levels of absenteeism.	☐ Mild symptoms and little or no associated disability<br>☐ Moderate symptoms and some disability<br>☐ Significantly disabling, associated with mortality risk or no effective treatments available

That it addresses an area of high social burden	LBP is the greatest cause of disability globally (YLDs). Worldwide prevalence of LBP is 9.4% (Damian Hoy et al., 2014) and this figure is increasing yearly with an aging population and rising obesity. The Australian prevalence of back pain is higher, with 3.7 million people (16%) effected in the period 2014-15 (Australian Institute of Health and Welfare, 2017).<br><br>Total direct and indirect costs of LBP are estimated to have cost AU$9.17 billion in 2001 (Walker, Muller, & Grant, 2003) and NZ$3 billion in 2015 (National Health Committee, 2015) in AU and NZ respectively. The substantial economic burden is carried by the government, employers, insurers, and individuals.	☐ Condition is rare<br>☐ Condition is somewhat common<br>☐ Condition is common<br>☐ Condition is highly prevalent
Potential reduction in patient and/or social burden due to (clinical or implementation) intervention	LBP appears to be a long term condition in both acute and chronic patients. In 85-95% of case there is no known cause and remission from LBP is rare. In most cases, individuals will go on to have frequent recurrent episodes that may worsen in intensity and duration. Recurrence rates vary between studies, with 12-month recurrent episode occurring in 30% to 80% of patients (D. Hoy et al., 2010). Until understanding of LBP improves, it is likely that interventions will address symptoms only.<br><br>There is very limited evidence regarding the effectiveness of treatment of LBP, with exercise and multimodal interventions performing better than GP care (Van Tulder, Malmivaara, Esmail, & Koes, 2000). There is overwhelming support suggesting that a return to work is associated with favourable outcomes (New Zealand Guidelines Group, 2004). If workplace interventions are show to be effective then promoting return to work alongside an effective care programme may be powerful combination in treating LBP.	☐ Symptomatic treatment only and small potential effect size<br>☐ Symptomatic treatment and large potential effect size<br>☐ Potential for intervention to treat both symptoms and underlying disease pathology<br>☐ Potential for cure or fundamental alteration of disease course
Potential scalability and uptake or intervention	Studies examining workplace interventions show evidence that exercise and multidisciplinary programs may have an effect on LBP (Tveito, Hysing, & Eriksen, 2004). As seen in the popularity of Los Angeles Lift Off study (Yancey et al., 2004) – a workplace physical activity program – uptake of an exercise or intervention program to manage LBP may prove to be highly popular and easy to scale. Many workplace behaviour change programmes depend on buy-in from management to be truly successful. When considering the burden associated with workplace absenteeism, an effective programme may be welcomed by companies to adopt in a top-down approach.	☐ Low potential for scalability and uptake<br>☐ High potential for uptake but low scalability<br>☐ High potential for scalability but low potential for uptake<br>☐ High potential for both scalability and uptake

Extent to which the question addresses health equity	Disability experienced is higher in low-income and middle-income countries (Clark & Horton, 2018) where access to health services is poor. This is problematic in populations with high levels of manual labour where the ability to take time off work is difficult and the risk of injury is higher. These populations also face a greater risk of LBP secondary to disease such as tuberculosis and undiagnosed malignancies (Hartvigsen et al., 2018). Similarities may be seen in Australia with a greater prevalence of LBP found in areas of most depravation (Australian Institute of Health and Welfare, 2017) where the likelihood of blue-collar employment is higher and access to healthcare is limited.	☐ No attempt to address health equity<br>☐ Discussed using Progress Plus items (O'Neill et al., 2014) but intervention not relevant or appropriate<br>☐ Intervention shown to have some potential application to improving health equity issues<br>☐ The intervention is explicitly designed to improve health equity issues

Final question:

In your opinion, do you think this research question is important enough for ANZMUSC to endorse it (assuming all other endorsement criteria are met)?

☐ Yes

☐ No

References

Australian Institute of Health and Welfare. (2017). *The Burden of Musculoskeletal Conditions in Australia - A Detailed Analysis of the Australian Burden of Disease Study 2011*. Retrieved from www.aihw.gov.au

Briggs, A. M., & Buchbinder, R. (2009). Back pain: a National Health Priority Area in Australia? *The Medical Journal of Australia*, *190*(9), 499–502. Retrieved from www.mja.com.au

Clark, S., & Horton, R. (2018). Low back pain: a major global challenge. *The Lancet*, *391*(10137), 2302. https://doi.org/10.1016/S0140-6736(18)30725-6

Costa, L. D. C. M., Koes, B. W., Pransky, G., Borkan, J., Maher, C. G., & Smeets, R. J. E. M. (2013). Primary care research priorities in Low Back Pain: An update. *Spine*, *38*(2), 148–156. https://doi.org/10.1097/BRS.0b013e318267a92f

Hartvigsen, J., Hancock, M. J., Kongsted, A., Louw, Q., Ferreira, M. L., Genevay, S., … Woolf, A. (2018). What low back pain is and why we need to pay attention. *The Lancet*. https://doi.org/10.1016/S0140-6736(18)30480-X

Hoy, D., Brooks, P., Blyth, F., & Buchbinder, R. (2010, December 1). The Epidemiology of low back pain. *Best Practice and Research: Clinical Rheumatology*. Baillière Tindall. https://doi.org/10.1016/j.berh.2010.10.002

Hoy, D., March, L., Brooks, P., Blyth, F., Woolf, A., Bain, C., … Buchbinder, R. (2014). The global burden of low back pain: Estimates from the Global Burden of Disease 2010 study. *Annals of the Rheumatic Diseases*, *73*(6), 968–974. https://doi.org/10.1136/annrheumdis-2013-204428

Mathy, C., Cedraschi, C., Broonen, J. P., Azzi, A., & Henrotin, Y. (2015). Volition and low back pain: When patients talk. *Annals of Physical and Rehabilitation Medicine*, *58*(6), 326–331. https://doi.org/10.1016/j.rehab.2015.10.005

Ministry of Health. (2016). *Health Loss in New Zealand 1990-2013: A report from the New Zealand Burden of*

Diseases, Injuries and Risk Factors Study. Wellington. Retrieved from https://www.health.govt.nz/publication/health-loss-new-zealand-1990-2013

National Health Committee. (2015). *National Health Committee - Low Back Pain: A Pathway to Prioritisation*. Wellington. Retrieved from https://www.health.govt.nz/system/files/documents/publications/nhc-lbp-pathway-to-prioritisation.pdf

New Zealand Guidelines Group. (2004). New Zealand Acute Low Back Pain Guide. *Low Back Pain Guidelines*.

Tveito, T. H., Hysing, M., & Eriksen, H. R. (2004). Low back pain interventions at the workplace: A systematic literature review. *Occupational Medicine*, *54*(1), 3–13. https://doi.org/10.1093/occmed/kqg109

Van Tulder, M., Malmivaara, A., Esmail, R., & Koes, B. (2000). Exercise therapy for low back pain: A systematic review within the framework of the cochrane collaboration back review group. *Spine*. https://doi.org/10.1097/00007632-200011010-00011

Walker, B. F. F., Muller, R., & Grant, W. D. D. (2003). Low back pain in Australian adults: The economic burden. *Asia-Pacific Journal of Public Health*, *15*(2), 79–87. https://doi.org/10.1177/101053950301500202

Widanarko, B., Legg, S., Stevenson, M., Devereux, J., Eng, A., ’t Mannetje, A., … Pearce, N. (2012). Prevalence and work-related risk factors for reduced activities and absenteeism due to low back symptoms. *Applied Ergonomics*, *43*(4), 727–737. https://doi.org/10.1016/j.apergo.2011.11.004

Yancey, A. K., McCarthy, W. J., Taylor, W. C., Merlo, A., Gewa, C., Weber, M. D., & Fielding, J. E. (2004). The Los Angeles Lift Off: a sociocultural environmental change intervention to integrate physical activity into the workplace. *Preventive Medicine*, *38*(6), 848–856. https://doi.org/10.1016/j.ypmed.2003.12.019

Research question template

The research question is:

Is addition of leflunomide to standard corticosteroid therapy better than standard corticosteroid therapy in patients with giant cell arteritis (GCA) in terms of reduction of steroid dose and quality of life?

Please tick one box for each of the following categories:

Category	Content relevant to this research question	Your rating of this category
Extent to which the question is important to patients and other health decision-makers	Patient priorities for the treatment are primarily focused on the long term risk and side-effects of oral corticosteroids (Liddle et al., 2017; Muller et al., 2018). Certainly finding a more effective therapy that allows for lower steroid dosage and faster tapering would be welcomed by patients. Clinicians also prioritise more effective therapies for GCA, particularly when fears exist around saving patient vision or preventing ischaemic complications. Several attempts to identify effective steroid-sparing treatments have failed and research in this area has slowed (Ponte, Rodrigues, O'Neill, & Luqmani, 2015).	☐ Not shown to be important to either patients or decision-makers<br>☐ Shown to be important to health decision-makers but not patients<br>☐ Shown to be important to patients but not decision-makers<br>☐ Shown to be important to both patients and decision-makers
That it addresses an area of high patient burden	Common symptoms stem from ischaemic and inflammatory processes and include a new headache, jaw claudication and scalp tenderness. Polymyalgia rheumatica is frequently associated with GCA resulting in systemic inflammatory symptoms such as pain, malaise, and weight loss. Serious complications exist requiring treatment without delay. Visual symptoms occur in roughly 30% of patients, with permanent visual loss occurring in ~15% of patients. Rarely, 2-4% of patients will experience stroke or myocardial infarction (Borchers & Gershwin, 2012).	☐ Mild symptoms and little or no associated disability<br>☐ Moderate symptoms and some disability<br>☐ Significantly disabling, associated with mortality risk or no effective treatments available
That it addresses an area of high social burden	Incidence around the world varies greatly where data are available. Risk is double in women compared to men, increases with age beyond 50 years old, and effects those in Scandinavian countries or with Scandinavian background the most. In Sweden, biopsy-confirmed incidence between 1976 and 1995 was 22.2/100,000 people per year (Petursdottir, Johansson, Nordborg, & Nordborg, 1999). An study of biopsy-confirmed Otago residents found incidence to be 12.7/100,000 annually (Abdul-Rahman, Molteno, & Bevin, 2011), and in South Australia the incidence is 3.2/100,000 annually (Dunstan et al., 2014).	☐ Condition is rare<br>☐ Condition is somewhat common<br>☐ Condition is common<br>☐ Condition is highly prevalent

Potential reduction in patient and/or social burden due to (clinical or implementation) intervention	While a genetic component has been identified, the cause of GCA is unknown and until more is known about the disease it is unlikely that any intervention will provide a cure. Due to a 50% chance of relapse during the 'taper down' period of treatment, patients often undergo treatment lasting a number of years (Fraser, Weyand, Newman, & Biousse, 2008). Adverse reactions to corticosteroid treatment for GCA will occur in up to 80% of people, with up to 38% experiencing fractures, and 41% experiencing cataracts (Proven, Gabriel, Orces, O'Fallon, & Hunder, 2003). There is poor evidence regarding the use of DMARD and corticosteroid-sparing treatment so continued research to identify new treatments to improve treatment outcomes may help to reduce side effects and recurrence of the disease.	☐ Symptomatic treatment only and small potential effect size<br>☐ Symptomatic treatment and large potential effect size<br>☐ Potential for intervention to treat both symptoms and underlying disease pathology<br>☐ Potential for cure or fundamental alteration of disease course
Potential scalability and uptake or intervention	Leflunomide is already widely used in New Zealand and Australia in the treatment of rheumatic conditions. Provided it is a safe and effective treatment alongside corticosteroids then its use could be easily implemented through appropriate training to prescribers. Early evidence suggests that strong side effects may mean the drug is not suitable for 1 in 4 users (Diamantopoulos, Hetland, & Myklebust, 2013) and frequent check-ups will be required during an introductory period.	☐ Low potential for scalability and uptake<br>☐ High potential for uptake but low scalability<br>☐ High potential for scalability but low potential for uptake<br>☐ High potential for both scalability and uptake
Extent to which the question addresses health equity	In a study of GCA patients in the United Kingdom it was found that the most deprived had dramatically higher likelihood (OR 4.2) of developing ischaemic complications compared to the least deprived (Mackie et al., 2011). The authors suggest the likelihood of delay between symptoms and presentation. Although much is needed to improve access to health care in areas of deprivation, the potential for new treatments for GCA may help to slow or prevent the progression of disease in areas where blindness or stroke will create high social burden.	☐ No attempt to address health equity<br>☐ Discussed using Progress Plus items (O'Neill et al., 2014) but intervention not relevant or appropriate<br>☐ Intervention shown to have some potential application to improving health equity issues<br>☐ The intervention is explicitly designed to improve health equity issues

Final question:

In your opinion, do you think this research question is important enough for ANZMUSC to endorse it (assuming all other endorsement criteria are met)?

☐ Yes

☐ No

References

Abdul-Rahman, A. M., Molteno, A. C. B., & Bevin, T. H. (2011). The epidemiology of giant cell arteritis in Otago, New Zealand: A 9-year analysis. *New Zealand Medical Journal*, *124*(1329), 44–52. https://doi.org/10.1002/art.24459

Borchers, A. T., & Gershwin, M. E. (2012). Giant cell arteritis: A review of classification, pathophysiology, geoepidemiology and treatment. *Autoimmunity Reviews*, *11*(6–7), A544–A554. https://doi.org/10.1016/j.autrev.2012.01.003

Diamantopoulos, A. P., Hetland, H., & Myklebust, G. (2013). Leflunomide as a corticosteroid-sparing agent in giant cell arteritis and polymyalgia rheumatica: A case series. *BioMed Research International*, 120638. https://doi.org/10.1155/2013/120638

Dunstan, E., Lester, S. L., Rischmueller, M., Dodd, T., Black, R., Ahern, M., … Hill, C. L. (2014). Epidemiology of biopsy-proven giant cell arteritis in South Australia. *Internal Medicine Journal*, *44*(1), 32–39. https://doi.org/10.1111/imj.12293

Fraser, J. A., Weyand, C. M., Newman, N. J., & Biousse, V. (2008). The treatment of giant cell arteritis. *Reviews in Neurological Diseases*, *5*(3), 140–152. https://doi.org/10.1016/j.bbi.2008.05.010

Liddle, J., Bartlam, R., Mallen, C. D., Mackie, S. L., Prior, J. A., Helliwell, T., & Richardson, J. C. (2017). What is the impact of giant cell arteritis on patients' lives? A UK qualitative study. *BMJ Open*, *7*(8), e017073. https://doi.org/10.1136/bmjopen-2017-017073

Mackie, S. L., Dasgupta, B., Hordon, L., Gough, A., Green, M., Hollywood, J., … UK GCA Consortium. (2011). Ischaemic manifestations in giant cell arteritis are associated with area level socio-economic deprivation, but not cardiovascular risk factors. *Rheumatology (Oxford, England)*, *50*(11), 2014–2022. https://doi.org/10.1093/rheumatology/ker265

Muller, S., Brien, A. O., Helliwell, T., Hay, C. A., Gilbert, K., & Mallen, C. D. (2018). Support available for and perceived priorities of people with polymyalgia rheumatica and giant cell arteritis : results of the PMRGCAuk members ' survey 2017. *Clinical Rheumatology*, 1–8. https://doi.org/https://doi.org/10.1007/s10067-018-4220-1

Petursdottir, V., Johansson, H., Nordborg, E., & Nordborg, C. (1999). The epidemiology of biopsy-positive giant cell arteritis: Special reference to cyclic fluctuations. *Rheumatology*, *38*(12), 1208–1212. https://doi.org/10.1093/rheumatology/38.12.1208

Ponte, C., Rodrigues, A. F., O'Neill, L., & Luqmani, R. A. (2015). Giant cell arteritis: Current treatment and management. *World Journal of Clinical Cases*, *3*(6), 484. https://doi.org/10.12998/wjcc.v3.i6.484

Proven, A., Gabriel, S. E., Orces, C., O'Fallon, W. M., & Hunder, G. G. (2003). Glucocorticoid therapy in giant cell arteritis: Duration and adverse outcomes. *Arthritis & Rheumatism*, *49*(5), 703–708. https://doi.org/10.1002/art.11388

Research question template

The research question is:

In femoroacetabular impingement syndrome (FAI syndrome) what is the impact of arthroscopic surgery for hip impingement compared with best conservative care?

Please tick one box for each of the following categories:

Category	Content relevant to this research question	Your rating of this category
Extent to which the question is important to patients and other health decision-makers	There is little evidence regarding patient expectations for the treatment of FAI. While the use of arthroscopy is popular amongst surgeons, it appears that there quality supporting evidence is lacking. A survey of 900 orthopaedic surgeons concluded that the evidence for diagnosis and treatment of FAI is perceived to be weak and that there was confusion over indications for arthroscopic management (Khan et al., 2016). Researchers show concern over the popularity and dramatic increase in use in the US, UK and AU against a lack of evidence justifying the use of hip arthroscopy (Cicuttini, Teichtahl, & Wang, 2017; Reiman & Thorborg, 2015). This may be underlie recent policy changes applied to Medicaid funding for hip arthroscopy in Australia (Department of Health, 2016).	☐ Not shown to be important to either patients or decision-makers<br>☐ Shown to be important to health decision-makers but not patients<br>☐ Shown to be important to patients but not decision-makers<br>☐ Shown to be important to both patients and decision-makers
That it addresses an area of high patient burden	The primary complaint of activity-dependent hip or groin pain, catching, stiffness, and giving-way that can result in severe discomfort and impact in activities of daily living (Griffin et al., 2016).<br><br>As well as involvement in the development of labral tears (Beck, 2005), FAI is a precursor to development of osteoarthritis of the hip and early detection and treatment may help to prevent progression (Agricola et al., 2013).	☐ Mild symptoms and little or no associated disability<br>☐ Moderate symptoms and some disability<br>☐ Significantly disabling, associated with mortality risk or no effective treatments available
That it addresses an area of high social burden	FAI producing symptoms appears to be rare. Radiological findings of hip morphology do not correlate well with symptomatic FAI presentation. Large cohort studies using radiography or MRI suggest that the prevalence of cam or pincer type morphologies is between 15% and 20% in asymptomatic adults (Pun, Kumar, & Lane, 2015). Occurring predominantly in males, the prevalence may be as high as 70% in semi-professional and professional athletes (Zadpoor, 2015).	☐ Condition is rare<br>☐ Condition is somewhat common<br>☐ Condition is common<br>☐ Condition is highly prevalent

Potential reduction in patient and/or social burden due to (clinical or implementation) intervention	Unlike the symptomatic control found in conservative care or physical therapy management, hip arthroscopy offers the potential for cure through modification of bony spurs, the femoral head and acetabulum, as well as repair to labrum or articular cartilage as a result of the bony morphology found in FAI.<br><br>As a predisposing factor for the development of osteoarthritis of the hip, early treatment using this minimally invasive treatment may have great long term effects. Importantly, there is very little evidence for the long term outcomes of hip arthroscopy, particularly when compared to conservative management, and further research is required.	☐ Symptomatic treatment only and small potential effect size<br>☐ Symptomatic treatment and large potential effect size<br>☐ Potential for intervention to treat both symptoms and underlying disease pathology<br>☐ Potential for cure or fundamental alteration of disease course
Potential scalability and uptake or intervention	Hip arthroscopy is considered to be a moderately difficult procedure with a high learning-curve (Hoppe et al., 2014). This is a likely reason that this surgery is predominantly performed by experienced surgeons and in developed nations (Khan et al., 2016). Despite its difficulty, this type of surgery has seen steady increase worldwide in recent years suggesting that with appropriate training and experience it has good scalability.	☐ Low potential for scalability and uptake<br>☐ High potential for uptake but low scalability<br>☐ High potential for scalability but low potential for uptake<br>☐ High potential for both scalability and uptake
Extent to which the question addresses health equity	There is little or no evidence to support that the development of FAI is impacted by social determinants of health or that any treatment will have any substantive effect in any particular groups of people.	☐ No attempt to address health equity<br>☐ Discussed using Progress Plus items (O'Neill et al., 2014) but intervention not relevant or appropriate<br>☐ Intervention shown to have some potential application to improving health equity issues<br>☐ The intervention is explicitly designed to improve health equity issues

Final question:

In your opinion, do you think this research question is important enough for ANZMUSC to endorse it (assuming all other endorsement criteria are met)?

☐ Yes

☐ No

References

Agricola, R., Heijboer, M. P., Bierma-Zeinstra, S. M. A., Verhaar, J. A. N., Weinans, H., & Waarsing, J. H. (2013). Cam impingement causes osteoarthritis of the hip: a nationwide prospective cohort study (CHECK). *Annals of the Rheumatic Diseases*, *72*(6), 918–923. https://doi.org/10.1136/annrheumdis-2012-201643

Beck, M. (2005). Hip morphology influences the pattern of damage to the acetabular cartilage. *Journal of Bone and Joint Surgery - British Volume*, *87–B*(7), 1012–1018. https://doi.org/10.1302/0301-620X.87B7.15203

Cicuttini, F. M., Teichtahl, A. J., & Wang, Y. (2017). Hip arthroscopy for femoroacetabular impingement: Use escalating beyond the evidence. *Medical Journal of Australia*. https://doi.org/10.5694/mja16.00821

Department of Health. (2016). *Medicare Benefits Schedule - new and amended listings*. Retrieved from https://www.health.gov.au/internet/budget/publishing.nsf/Content/43F6C9859B1FEAA9CA257FA80010E5CB/$File/09_FS_MBS_NewAmended_1May_PRINT.pdf

Griffin, D. R., Dickenson, E. J., O'donnell, J., Agricola, R., Awan, T., Beck, M., & Clohisy, J. C. (2016). The Warwick Agreement on femoroacetabular impingement syndrome (FAI syndrome): an international consensus statement. *British Journal of Sports Medicine*, *50*(19), 1169–1176. https://doi.org/10.1136/bjsports-2016-096743

Hoppe, D. J., de SA, D., Simunovic, N., Bhandari, M., Safran, M. R., Larson, C. M., & Ayeni, O. R. (2014). The Learning Curve for Hip Arthroscopy: A Systematic Review. *Arthroscopy: The Journal of Arthroscopic & Related Surgery*, *30*(3), 389–397. https://doi.org/10.1016/J.ARTHRO.2013.11.012

Khan, M., Ayeni, O. R., Madden, K., Bedi, A., Ranawat, A., Kelly, B. T., … Bhandari, M. (2016). Femoroacetabular Impingement: Have We Hit a Global Tipping Point in Diagnosis and Treatment? Results From the InterNational Femoroacetabular Impingement Optimal Care Update Survey (IN FOCUS). *Arthroscopy: The Journal of Arthroscopic & Related Surgery*, *32*(5), 779–787.e4. https://doi.org/10.1016/J.ARTHRO.2015.10.011

Pun, S., Kumar, D., & Lane, N. E. (2015). Femoroacetabular Impingement. *Arthritis Rheumatol*, *67*(1), 17–27. https://doi.org/10.1002/art.38887.Femoroacetabular

Reiman, M. P., & Thorborg, K. (2015). Femoroacetabular impingement surgery: are we moving too fast and too far beyond the evidence? *British Journal of Sports Medicine*, *49*(12), 782–784. https://doi.org/10.1136/bjsports-2014-093821

Zadpoor, A. A. (2015). Etiology of Femoroacetabular Impingement in Athletes: A Review of Recent Findings. *Sports Medicine*, *45*(8), 1097–1106. https://doi.org/10.1007/s40279-015-0339-2

Research question template

The research question is:

Are remotely-delivered physiotherapist consultations as effective as in-clinic consultations for improving pain and function in people with knee osteoarthritis?

Please tick one box for each of the following categories:

Category	Content relevant to this research question	Your rating of this category
Extent to which the question is important to patients and other health decision-makers	Physiotherapy is considered relevant by people living in rural and remote areas, and is not always easily accessible (Cottrell, Hill, O'Leary, Raymer, & Russell, 2018). Exercise interventions, often delivered by physiotherapy, have been shown to improve pain and function in people with knee OA.	☐ Not shown to be important to either patients or decision-makers<br>☐ Shown to be important to health decision-makers but not patients<br>☐ Shown to be important to patients but not decision-makers<br>☐ Shown to be important to both patients and decision-makers
That it addresses an area of high patient burden	Knee osteoarthritis is associated with a broad spectrum of pain and disability, from minimal or no symptoms to marked loss of function, pain and psychological distress. There is no associated mortality risk. In Australia, osteoarthritis accounts for approximately 2% of the burden of disease, resulting in 83,405 DALYs in 2011. Similarly, the burden of disease is increasing in New Zealand and OA accounted for approximately 1% (10,000 DALY) in 2013.	☐ Mild symptoms and little or no associated disability<br>☐ Moderate symptoms and some disability<br>☐ Significantly disabling, associated with mortality risk or no effective treatments available
That it addresses an area of high social burden	Osteoarthritis of the hip and knee was ranked 11th highest global contributor to disability, with a prevalence of 3.8% and 0.85% for knee and hip respectively (Cross et al., 2014). In Australia the age-adjusted prevalence of diagnosed osteoarthritis is 8.1% (Australian Institute of Health and Welfare, 2018) and in New Zealand it is 7.2% (Ministry of Health, 2017). With substantially higher incidences occurring in women and those aged over 65, these figures are set to increase due to the rising levels of obesity and an aging population.	☐ Condition is rare<br>☐ Condition is somewhat common<br>☐ Condition is common<br>☐ Condition is highly prevalent

Potential reduction in patient and/or social burden due to (clinical or implementation) intervention	Interventions provided by physiotherapy, particularly exercise has been shown to improve pain and function with small to moderate effect sizes in the short to medium term for knee OA (Fransen et al., 2015).	☐ Symptomatic treatment only and small potential effect size<br>☐ Symptomatic treatment and large potential effect size<br>☐ Potential for intervention to treat both symptoms and underlying disease pathology<br>☐ Potential for cure or fundamental alteration of disease course
Potential scalability and uptake or intervention	Technology that enables tele-health interventions is widely available and getting cheaper. However, internet access in rural and remote Australia is not as easily available as in metropolitan areas. The Australian Medical Association has called for better rural access to high speed broadband internet (https://ama.com.au/position-statement/better-access-high-speed-broadband-rural-and-remote-health-care-2016).	☐ Low potential for scalability and uptake<br>☐ High potential for uptake but low scalability<br>☐ High potential for scalability but low potential for uptake<br>☐ High potential for both scalability and uptake
Extent to which the question addresses health equity	Increasing access to an effective intervention is a key objective of this research proposal. Using the PROGRESS approach, tele-health and remotely delivered interventions to people living in rural and remote areas address equity issues concerning place (geographical isolation), race/ethnicity (greater proportion of indigenous people), and socioeconomic status (greater proportion of socioeconomic deprivation).	☐ No attempt to address health equity<br>☐ Discussed using Progress Plus items (O'Neill et al., 2014) but intervention not relevant or appropriate<br>☐ Intervention shown to have some potential application to improving health equity issues<br>☐ The intervention is explicitly designed to improve health equity issues

Final question:

In your opinion, do you think this research question is important enough for ANZMUSC to endorse it (assuming all other endorsement criteria are met)?

☐ Yes

☐ No

References

Cottrell, M. A., Hill, A. J., O'Leary, S. P., Raymer, M. E., & Russell, T. G. (2018). Patients are willing to use telehealth for the multidisciplinary management of chronic musculoskeletal conditions: A cross-sectional survey. *Journal of Telemedicine & Telecare, 24*(7), 445-452. doi:https://dx.doi.org/10.1177/1357633X17706605

Fransen, M., McConnell, S., Harmer, A. R., Van der Esch, M., Simic, M., & Bennell, K. L. (2015). Exercise for osteoarthritis of the knee. *Cochrane Database of Systematic Reviews*(1). doi:10.1002/14651858.CD004376.pub3

Research question template

The research question is:

Is a deprescribing intervention effective in reducing opioid use in people with chronic low back pain?

Please tick one box for each of the following categories:

Category	Content relevant to this research question	Your rating of this category
Extent to which the question is important to patients and other health decision-makers	Both patients and clinicians have concerns regarding the use of opioids for chronic non cancer pain. Patient fears over the addictive nature of opioids and being associated with non-pain opioid users (Smith et al., 2015). Highly experienced clinicians appear to have greater hesitance in prescribing and are aware of the importance of reducing or discontinuing use when dependency behaviours are present (Razouki, Khokhar, Philpot, & Ebbert, 2018).	☐ Not shown to be important to either patients or decision-makers<br>☐ Shown to be important to health decision-makers but not patients<br>☐ Shown to be important to patients but not decision-makers<br>☐ Shown to be important to both patients and decision-makers
That it addresses an area of high patient burden	LBP is the biggest cause of disability worldwide (Hoy et al., 2014) and can be long lasting and debilitating requiring the use of analgesics to manage pain, such as opioids. Long term use of opioids can reduce drug efficacy, create dependency and in some cases addiction and overdose. Opioid dependency can develop in as little as 1 to 3 months of use with half of >3 month users continuing to use them for more than 1 year (Deyo, Von Korff, & Duhrkoop, 2015). Opioid dependency accounts for 9.2M DALY's worldwide yearly, and increase of 73% since the 1990's (Degenhardt et al., 2014)	☐ Mild symptoms and little or no associated disability<br>☐ Moderate symptoms and some disability<br>☐ Significantly disabling, associated with mortality risk or no effective treatments available
That it addresses an area of high social burden	LBP is the greatest cause of disability globally (YLDs). Worldwide prevalence of LBP is 9.4% (Hoy et al., 2014). The Australian prevalence of back pain is higher, with 3.7 million people (16%) effected in the period 2014-15 (Australian Institute of Health and Welfare, 2017).<br>While opioid use appears to have little effect on outcome measures for low back pain (Deyo et al., 2015), their use has seen swelling uptake in recent years. In Australia, the use of morphine has dropped while the use of oxycodone has increased by 150% in the 6 years ending 2008 (Roxburgh, Bruno, Larance, & Burns, 2011) with nearly half prescribed for chronic non cancer pain (Harrison, Charles, Henderson, & Britt, 2012). In Australia 3% of the population report using opioids for non-medical reasons (Australian Institute of Health and Welfare, 2013).	☐ Condition is rare<br>☐ Condition is somewhat common<br>☐ Condition is common<br>☐ Condition is highly prevalent

Potential reduction in patient and/or social burden due to (clinical or implementation) intervention	Very few studies have examined the effectiveness of opioids for back pain compared to non-placebo treatment and many opioid studies are prone to bias (Deyo et al., 2015). As the use of opioids appears unlikely to improve long term outcome measures for back pain, any benefits in pain management seen by individuals are likely to be small.	☐ Symptomatic treatment only and small potential effect size<br>☐ Symptomatic treatment and large potential effect size<br>☐ Potential for intervention to treat both symptoms and underlying disease pathology<br>☐ Potential for cure or fundamental alteration of disease course
Potential scalability and uptake or intervention	There is limited evidence beyond expert guidelines regarding deprescription protocols. Prescriber and dispensary continuity is essential and a registry might be required to control prescriptions. Prescription monitoring and reduced pill count at higher frequencies to limit access may be useful .Close supervision of patients, depending on levels of dependency or those showing risk behaviours, is required. Urine sampling may be useful as self-reporting is not usually accurate. (Huntzinger, 2009)<br>With good education it is likely that the majority of opioid users would be willing to reduce usage, with only a small population of users showing risk behaviours such as abuse or diversion.	☐ Low potential for scalability and uptake<br>☐ High potential for uptake but low scalability<br>☐ High potential for scalability but low potential for uptake<br>☐ High potential for both scalability and uptake
Extent to which the question addresses health equity	Disability as a result of low back pain is higher in Australian areas of most depravation (Australian Institute of Health and Welfare, 2017) where males living with unemployment or homelessness are more likely to be illicit opioid users (Degenhardt et al., 2006). Providing pathways to reduce opioid use may help support users to<br>Indigenous Australians are 3 times more likely to receive pharmacotherapy for treatment of opioid dependency (Australian Institute of Health and Welfare, 2013).	☐ No attempt to address health equity<br>☐ Discussed using Progress Plus items (O'Neill et al., 2014) but intervention not relevant or appropriate<br>☐ Intervention shown to have some potential application to improving health equity issues<br>☐ The intervention is explicitly designed to improve health equity issues

Final question:

In your opinion, do you think this research question is important enough for ANZMUSC to endorse it (assuming all other endorsement criteria are met)?

☐ Yes

☐ No

References

Australian Institute of Health and Welfare. (2013). *National opioid pharmacotherapy statistics annual data (NOPSAD) collection 2012*. Canberra. Retrieved from https://www.aihw.gov.au/reports/alcohol-other-drug-treatment-services/nopsad-2012/contents/table-of-contents

Australian Institute of Health and Welfare. (2017). *The Burden of Musculoskeletal Conditions in Australia - A Detailed Analysis of the Australian Burden of Disease Study 2011*. Retrieved from www.aihw.gov.au

Degenhardt, L., Black, E., Breen, C., Bruno, R., Kinner, S., Roxburgh, A., … Fischer, J. (2006). Trends in morphine prescriptions, illicit morphine use and associated harms among regular injecting drug users in Australia. *Drug and Alcohol Review*, *25*(5), 403–412. https://doi.org/10.1080/09595230600868504

Degenhardt, L., Charlson, F., Mathers, B., Hall, W. D., Flaxman, A. D., Johns, N., & Vos, T. (2014). The global epidemiology and burden of opioid dependence: Results from the global burden of disease 2010 study. *Addiction*, *109*(8), 1320–1333. https://doi.org/10.1111/add.12551

Deyo, R. A., Von Korff, M., & Duhrkoop, D. (2015). Opioids for low back pain. *BMJ (Online)*, *350*. https://doi.org/10.1136/bmj.g6380

Harrison, C. M., Charles, J., Henderson, J., & Britt, H. (2012). Opioid prescribing in Australian general practice. *Medical Journal of Australia*, *196*(6), 380–381. https://doi.org/10.5694/mja12.10168

Hoy, D., March, L., Brooks, P., Blyth, F., Woolf, A., Bain, C., … Buchbinder, R. (2014). The global burden of low back pain: Estimates from the Global Burden of Disease 2010 study. *Annals of the Rheumatic Diseases*, *73*(6), 968–974. https://doi.org/10.1136/annrheumdis-2013-204428

Huntzinger, A. (2009). Guidelines for the use of opioid therapy in patients with chronic noncancer pain. *American Family Physician*, *80*(11), 1315–1316. https://doi.org/10.1016/j.jpain.2008.10.008

Razouki, Z., Khokhar, B. A., Philpot, L. M., & Ebbert, J. O. (2018). Attributes, Attitudes, and Practices of Clinicians Concerned with Opioid Prescribing. *Pain Medicine*. https://doi.org/10.1093/pm/pny204

Roxburgh, A., Bruno, R., Larance, B., & Burns, L. (2011). Prescription of opioid analgesics and related harms in Australia. *Medical Journal of Australia*, *195*(5), 280–284. https://doi.org/10.5694/mja10.11450

Smith, R. J., Rhodes, K., Paciotti, B., Kelly, S., Perrone, J., & Meisel, Z. F. (2015). Patient Perspectives of Acute Pain Management in the Era of the Opioid Epidemic. *Annals of Emergency Medicine*, *66*(3), 246–252. https://doi.org/10.1016/j.annemergmed.2015.03.025

Research question template

The research question is:

Is muscle strengthening exercise better than standard care for people with newly diagnosed inflammatory myopathy (myositis) in terms of pain and function at 6 months?

Please tick one box for each of the following categories:

Category	Content relevant to this research question	Your rating of this category
Extent to which the question is important to patients and other health decision-makers	Research investigating patient and clinician priorities for the management of inflammatory myositis are lacking. A small study examining patient preferences suggested that physical activities such as walking and cycling were important disability outcomes (Alemo Munters, van Vollenhoven, & Alexanderson, 2011).	☐ Not shown to be important to either patients or decision-makers<br>☐ Shown to be important to health decision-makers but not patients<br>☐ Shown to be important to patients but not decision-makers<br>☐ Shown to be important to both patients and decision-makers
That it addresses an area of high patient burden	Inflammatory myopathies are an autoimmune condition characterised by chronic muscle weakness. Patients in South Australia with idiopathic inflammatory myelitis have a 75% increase in risk of mortality due to cardiovascular, infection, diabetes, and malignancy (Limaye, Hakendorf, Woodman, Blumbergs, & Roberts-Thomson, 2012). Long term corticosteroid treatment can result in outcome such as vertebral fractures (Clarke, Bloch, Medsger, & Oddis, 1995).	☐ Mild symptoms and little or no associated disability<br>☐ Moderate symptoms and some disability<br>☐ Significantly disabling, associated with mortality risk or no effective treatments available
That it addresses an area of high social burden	Idiopathic inflammatory myopathies are a rare group of rheumatic autoimmune diseases consists of several subcategories. The incidence of this group worldwide varies geographically with an estimated incidence of 8 per million per year (Meyer et al., 2014) which matches the incidence in the South Australian population (Tan et al., 2013).	☐ Condition is rare<br>☐ Condition is somewhat common<br>☐ Condition is common<br>☐ Condition is highly prevalent

Potential reduction in patient and/or social burden due to (clinical or implementation) intervention	A series of recent studies have suggested beneficial outcomes following exercise in patients with myositis. It appears that regular exercise reduces the inflammatory marker serum creatine phosphokinase as well as downregulation of some gene expressions found in myositis (Alemo Munters, Alexanderson, Crofford, & Lundberg, 2014).	☐ Symptomatic treatment only and small potential effect size<br>☐ Symptomatic treatment and large potential effect size<br>☐ Potential for intervention to treat both symptoms and underlying disease pathology<br>☐ Potential for cure or fundamental alteration of disease course
Potential scalability and uptake or intervention	Exercise prescription for patients with inflammatory myositis can be incorporated in to regular physical therapy programmes. A home-based exercise programme appears to be safe and effective in managing the disease in early stages (Alexanderson, Stenström, Jenner, & Lundberg, 2000).	☐ Low potential for scalability and uptake<br>☐ High potential for uptake but low scalability<br>☐ High potential for scalability but low potential for uptake<br>☐ High potential for both scalability and uptake
Extent to which the question addresses health equity	Research investigating the health equity of patients with inflammatory myopathies is sparse. While indigenous populations such as First Nations, Australian aboriginals, and New Zealand Maori have a higher prevalence of rheumatic conditions (McDougall, Hurd, & Barnabe, 2017), there does not appear to be any data on inflammatory myopathies specifically.	☐ No attempt to address health equity<br>☐ Discussed using Progress Plus items (O'Neill et al., 2014) but intervention not relevant or appropriate<br>☐ Intervention shown to have some potential application to improving health equity issues<br>☐ The intervention is explicitly designed to improve health equity issues

Final question:

In your opinion, do you think this research question is important enough for ANZMUSC to endorse it (assuming all other endorsement criteria are met)?

☐ Yes

☐ No

References

Alemo Munters, L., Alexanderson, H., Crofford, L. J., & Lundberg, I. E. (2014). New insights into the benefits of exercise for muscle health in patients with idiopathic inflammatory myositis. *Current Rheumatology Reports*, *16*(7), 429. https://doi.org/10.1007/s11926-014-0429-4

Alemo Munters, L., van Vollenhoven, R. F., & Alexanderson, H. (2011). Patient Preference Assessment Reveals Disease Aspects Not Covered by Recommended Outcomes in Polymyositis and Dermatomyositis. *ISRN Rheumatology*, *2011*, 1–5. https://doi.org/10.5402/2011/463124

Alexanderson, H., Stenström, C. H., Jenner, G., & Lundberg, I. (2000). The safety of a resistive home exercise program in patients with recent onset active polymyositis or dermatomyositis. *Scandinavian Journal of Rheumatology*, *29*(5), 295–301. https://doi.org/10.1080/030097400447679

Clarke, A. E., Bloch, D. A., Medsger, T. A., & Oddis, C. V. (1995). A longitudinal study of functional disability in a national cohort of patients with polymyositis/dermatomyositis. *Arthritis & Rheumatism*, *38*(9), 1218–1224. https://doi.org/10.1002/art.1780380907

Limaye, V., Hakendorf, P., Woodman, R. J., Blumbergs, P., & Roberts-Thomson, P. (2012). Mortality and its predominant causes in a large cohort of patients with biopsy-determined inflammatory myositis. *Internal Medicine Journal*, *42*(2), 191–198. https://doi.org/10.1111/j.1445-5994.2010.02406.x

McDougall, C., Hurd, K., & Barnabe, C. (2017). Systematic review of rheumatic disease epidemiology in the indigenous populations of Canada, the United States, Australia, and New Zealand. *Seminars in Arthritis and Rheumatism*, *46*(5), 675–686. https://doi.org/10.1016/j.semarthrit.2016.10.010

Meyer, A., Meyer, N., Schaeffer, M., Gottenberg, J. E., Geny, B., & Sibilia, J. (2014). Incidence and prevalence of inflammatory myopathies: A systematic review. *Rheumatology (United Kingdom)*, *54*(1), 50–63. https://doi.org/10.1093/rheumatology/keu289

Tan, J. A., Roberst-Thomson, P. J., Blumbergs, P., Hakendorf, P., Cox, S. R., & Limaye, V. (2013). Incidence and prevalence of idiopathic inflammatory myopathies in South Australia: a 30-year epidemiologic study of histology-proven cases. *International Journal of Rheumatic Diseases*, *16*(3), 331–338. https://doi.org/10.1111/j.1756-185X.2011.01669.x

Research question template

The research question is:

Does red flag evaluation improve management of musculoskeletal disorders?

Please tick one box for each of the following categories:

Category	Content relevant to this research question	Your rating of this category
Extent to which the question is important to patients and other health decision-makers	Red flag screening is common place amongst clinicians and is often found in published clinical guidelines. Despite poor supporting evidence (Williams et al., 2013), there is little or no research suggesting that clinicians or decision-makers see this as an important priority.	☐ Not shown to be important to either patients or decision-makers<br>☐ Shown to be important to health decision-makers but not patients<br>☐ Shown to be important to patients but not decision-makers<br>☐ Shown to be important to both patients and decision-makers
That it addresses an area of high patient burden	Many serious pathological process' have common musculoskeletal presentation which can represent dangerous underlying conditions. For example, misdiagnosed back pain caused by cauda equina syndrome can result in devastating loss of bladder, bowel and sexual function (Lavy, James, Wilson-MacDonald, & Fairbank, 2009). Giant Cell Arteritis presenting as a headache or jaw pain can result in permanent vision loss or stroke if not correctly identified (Reiter, Winocur, Goldsmith, Emodi-Perlman, & Gorsky, 2009). Red flag screening can potentially identify these pathologies quickly so that potential life threatening outcomes can be avoided.	☐ Mild symptoms and little or no associated disability<br>☐ Moderate symptoms and some disability<br>☐ Significantly disabling, associated with mortality risk or no effective treatments available
That it addresses an area of high social burden	Red flag screening is a tool used by clinicians to ensure patient presentation is not due to underlying pathology. Usually these presentations are rare; in patients with low back pain, underlying pathologies account for less than 1% or presentations (Slipman et al., 2003; van den Bosch, Hollingworth, Kinmonth, & Dixon, 2004). However, it is crucial that these rare cases are identified and treated correctly. When given hypothetic cases, physical therapists made the correct decision regarding appropriate care for medically critical cases 79% of the time and were more likely to make the correct decision if they had specialist training (Jette, Ardleigh, Chandler, & McShea, 2006). Development of more robust red flag screening protocols may help in the correct diagnosis in these critical cases.	☐ Condition is rare<br>☐ Condition is somewhat common<br>☐ Condition is common<br>☐ Condition is highly prevalent

Potential reduction in patient and/or social burden due to (clinical or implementation) intervention	More and more patients are visiting their general practitioner, physiotherapist, or alternative healthcare provider for musculoskeletal symptoms. In the small proportion of cases where underlying pathology is the cause of pain, there is a real present risk for misdiagnosis and subsequent delay in appropriate receiving intervention. Effective red flag screening has the ability to make clinically relevant decisions regarding the care of patients, enabling the best levels of treatment for true musculoskeletal disorders, or rapid referral of a previously undiagnosed disease.	☐ Symptomatic treatment only and small potential effect size<br>☐ Symptomatic treatment and large potential effect size<br>☐ Potential for intervention to treat both symptoms and underlying disease pathology<br>☐ Potential for cure or fundamental alteration of disease course
Potential scalability and uptake or intervention	A review of screening for red flags for low back pain suggested that there is poor evidence to support the screening of many red flags currently examined (Williams et al., 2013) resulting in testing and under-treatment of patients (Henschke et al., 2013). Confirmation or rejection of the validity of red flag screening could be disseminated through clinician training and curriculum changes, however resistance to adoption of clinical guidelines as well as delays in implementation of research are well documented (Buchbinder, Maher, & Harris, 2015) and any change to current best-practices may take years to implement.	☐ Low potential for scalability and uptake<br>☐ High potential for uptake but low scalability<br>☐ High potential for scalability but low potential for uptake<br>☐ High potential for both scalability and uptake
Extent to which the question addresses health equity	Effective clinical screening processes for red flags are a simple way to identify patients that may be experiencing underlying pathology. A protocol may be developed for use by non-clinical personnel such as social workers in areas were healthcare access is limited. This may allow early identification of pathology in order to recommend those living in areas of deprivation seek medical care where this would not normally be done.	☐ No attempt to address health equity<br>☐ Discussed using Progress Plus items (O'Neill et al., 2014) but intervention not relevant or appropriate<br>☐ Intervention shown to have some potential application to improving health equity issues<br>☐ The intervention is explicitly designed to improve health equity issues

Final question:

In your opinion, do you think this research question is important enough for ANZMUSC to endorse it (assuming all other endorsement criteria are met)?

☐ Yes

☐ No

References

Buchbinder, R., Maher, C., & Harris, I. A. (2015). Setting the research agenda for improving health care in musculoskeletal disorders. *Nature Reviews Rheumatology*, *11*(10), 597–605. https://doi.org/10.1038/nrrheum.2015.81

Henschke, N., Maher, C. G., Ostelo, R. W. J. G., de Vet, H. C. W., Macaskill, P., & Irwig, L. (2013). Red flags to screen for malignancy in patients with low-back pain. *The Cochrane Database of Systematic Reviews*, *2*(2), CD008686. https://doi.org/10.1002/14651858.CD008686.pub2

Jette, D. U., Ardleigh, K., Chandler, K., & McShea, L. (2006). Decision-Making Ability of Physical Therapists: Physical Therapy Intervention or Medical Referral. *Physical Therapy*, *86*(12), 1619–1629. https://doi.org/10.2522/ptj.20050393

Lavy, C., James, A., Wilson-MacDonald, J., & Fairbank, J. (2009). Cauda equina syndrome. *BMJ*, *338*(mar31 1), b936–b936. https://doi.org/10.1136/bmj.b936

Reiter, S., Winocur, E., Goldsmith, C., Emodi-Perlman, A., & Gorsky, M. (2009). Giant cell arteritis misdiagnosed as temporomandibular disorder: a case report and review of the literature. *J Orofac Pain*, *23*(4), 360–365. Retrieved from https://s3.amazonaws.com/academia.edu.documents/31407841/giant_cell_arteritis_misdiagnosed_as_temporomandibular_disorder_a_case_report_and_review_of_the_literature_%282%29.pdf?AWSAccessKeyId=AKIAIWOWYYGZ2Y53UL3A&Expires=1541465825&Signature=E90XqQVGX0RFGk

Slipman, C. W., Patel, R. K., Botwin, K., Huston, C., Zhang, L., Lenrow, D., & Garvan, C. (2003). Epidemiology of spine tumors presenting to musculoskeletal physiatrists. *Archives of Physical Medicine and Rehabilitation*, *84*(4), 492–495. https://doi.org/10.1053/apmr.2003.50125

van den Bosch, M. A. A. J., Hollingworth, W., Kinmonth, A. L., & Dixon, A. K. (2004). Evidence against the use of lumbar spine radiography for low back pain. *Clinical Radiology*, *59*(1), 69–76. https://doi.org/10.1016/j.crad.2003.08.012

Williams, C. M., Henschke, N., Maher, C. G., van Tulder, M. W., Koes, B. W., Macaskill, P., & Irwig, L. (2013). Red flags to screen for vertebral fracture in patients presenting with low-back pain. *Cochrane Database of Systematic Reviews*, *2013*(1). https://doi.org/10.1002/14651858.CD008643.pub2
