# Supplementary material for: Which clinical research questions are the most important? Development and preliminary validation of the Australia & New Zealand Musculoskeletal (ANZMUSC) Clinical Trials Network Research Question Importance Tool (ANZMUSC-RQIT)
Source: PLoS One. 2023 Mar 17;18(3):e0281308. doi: 10.1371/journal.pone.0281308 (PMC10022765; doi:10.1371/journal.pone.0281308)
Supplement: S1 Table — (PDF) [file pone.0281308.s003.pdf]

S1 Table. Association of Cochrane Risk of Bias dimension categories with Journal Impact Factor group

**Risk of bias dimension**	**Risk of bias category***	**Low Impact journals (count of articles)**	**High Impact journals (count of articles)**	**Chi-square (2 df)**	**Cramer's V**	**p-value**
Randomisation	Low	80	97	14	0.27	<0.001
	Some	19	3			
	High	1	0			
Intervention deviation	Low	90	94	1.2	0.08	0.45
	Some	4	2			
	High	6	4			
Missing outcome data	Low	89	99	9.5	0.22	<0.002
	Some	3	1			
	High	8	0			
Measurement bias	Low	39	67	16	0.28	<0.001
	Some	58	32			
	High	3	11			
Selective reporting	Low	86	99	13	0.26	<0.001
	Some	2	1			
	High	12	0			
Overall risk of bias	Low	28	67	34	0.41	<0.001
	Some	47	28			

	High	25	5			

Low = low risk of bias, Some = some concerns, High = high risk of bias
