# Supplementary material for: Which clinical research questions are the most important? Development and preliminary validation of the Australia & New Zealand Musculoskeletal (ANZMUSC) Clinical Trials Network Research Question Importance Tool (ANZMUSC-RQIT)
Source: PLoS One. 2023 Mar 17;18(3):e0281308. doi: 10.1371/journal.pone.0281308 (PMC10022765; doi:10.1371/journal.pone.0281308)
Supplement: Attachment — Submitted filename: Response to reviewers_final.docx [file pone.0281308.s004.docx]

17 January 2022

Re: PONE-D-22-23846

Development and preliminary validation of the Australia & New Zealand Musculoskeletal (ANZMUSC) Clinical Trials Network Research Question Importance Tool (RQITv2.0)

Charlotte Beaudart

Academic Editor

PLoS One

Dear Dr Beaudart

Many thanks for allowing us the opportunity of re-submission. I have tabulated our response to the reviewers’ and editor’s comments. Any references to the text refer to the re-submitted version of the manuscript.

The funding statement should state:

This study was funded by the Australian National Health and Medical Research Council (NHMRC) Australia & New Zealand Musculoskeletal (ANZMUSC) Clinical Trials Network Centre of Research Excellence (APP1134856). RB is supported by an NHMRC Investigator Fellowship (APP1194483). RSH is supported by an NHMRC Senior Research Fellowship (APP1154217). The funders had no role in study design, data collection and analysis, decision to publish, or preparation of the manuscript.

I would also like to acknowledge the tragic and unexpected illness and death of one of the authors, Philip Robinson, during the preparation of the revision.

Yours sincerely,

William Taylor

|  | **Response** | **Changes to manuscript** |
| --- | --- | --- |
| **Editorial comments** |  |  |
| File naming and format requirements | We have re-named files and formatted the manuscript to conform to style requirements | Level 1 headings bold and 18pt font; level 2 headings bold and 16pt font, Figures and Table citations have been changed. Supplementary files have been created. |
| Mismatch of ‘funding information’ and ‘financial disclosure’ statements | Correct grants numbers are now provided |  |
| Funding statement | Amended funding statement to include all funding and sources of support, in cover letter above. |  |
| Acknowledgements section | Funding information moved to the Funding Statement as suggested. |  |
| Data availability | We have added further data files to the repository to permit a minimal data-set. |  |
| Group authorship | We wish to withdraw the group author as all authors are already individually included. | Author list is amended. |
|  |  |  |
| **Reviewer 1 comments** |  |  |
| Unclear title | We have changed the title to emphasize the context of the assessment tool. | Title is now changed from:  “Development and preliminary validation of the Australia & New Zealand Musculoskeletal (ANZMUSC) Clinical Trials Network Research Question Importance Tool (RQITv2.0)”  to:  “Which clinical research questions are the most important? Development and preliminary validation of the Australia & New Zealand Musculoskeletal (ANZMUSC) Clinical Trials Network Research Question Importance Tool” |
| The developers of this tool are all from Australia or New Zealand. If the goal is to provide a tool specifically for Australia and New Zealand, I wonder if this merits an international publication. | The goal is to provide a tool applicable in any geographic location. There are no aspects of the work that relate to local factors. | No changes made. |
| Naming of the tool version | We agree that this is potentially misleading, but we needed a means of referring to different versions of the tool as it developed during the study. | We have removed 2.0 from the name of the final version of the tool and have used a different naming system within the manuscript to facilitate accurate understanding. |
| MSK specific tool | We agree and have referred to the tool as Australia and New Zealand Musculoskeletal Clinical Trials Network Research Question Importance Tool (ANZMUSC-RQIT) to reflect this. | ANZMUSC appended to final tool name in title and throughout manuscript. |
| Validation | As noted in the limitations section of the manuscript, we agree that validation is challenging and bibliographic indices are far from ideal. However, the suggested sensitivity analysis will only compound this problem by continuing to employ bibliographic indices that will have even less certain meaning.  A key step in our approach was to confirm a relationship exists between clinical trial quality as assessed by the Cochrane RoB tool and journal impact factor (see page 15, line 316-318 in methods and page 26 lines 462-464 in results (and also Table S1). We believe this is a better way to provide evidence to support our approach. | No changes made. |
| Patient involvement | We are surprised by the reviewer’s comment and wonder if our use of the term ‘consumer’ was somehow misunderstood. We have therefore clarified in the manuscript that consumers included patients and described their involvement in more detail. | Additional text ln195-201 on pg 11:  “Consumers (health-service users) or consumer advocates were involved at every stage of the project, including generation of possible attributes of research importance (9% of participants), consensus development of the initial RQIT framework (18% of participants), weighting of specific elements of the framework using a novel best-work scaling method (12% of participants) and final authorship of this manuscript. Consumers were identified from membership of ANZMUSC and all had lived experience of musculoskeletal disorders.” |
| Hypothetical research questions | These are now provided as a supplementary appendix. We do not believe it is necessary to repeat the reliability analysis. It is sufficiently clear that scoring by individuals is not reliable and only scoring by committee consensus is adequately reliable. | New supplementary appendix. |
| Next steps for validation | This is a fair comment. The main next step for assessing the tool will come from actual use in practice. We thought it was important that the tool be made available for other groups to use by having the work published. We argue that it is sufficiently ready for practical use but that further evaluation will only come from experience in practical settings. That could not be part of the current development phase which is described in this manuscript. | Additional text in the Discussion section page 34, lines: 590-592  “Further validity evaluation should be undertaken by assessment of how the tool works prospectively in practice and comparison of scores with citations in subsequent clinical guidelines.” |
| Include the full final version and scoring instructions | This was already provided as Table 4 and a worked example was provided. | No changes made. |
|  |  |  |
| **Reviewer 2** |  |  |
| Global context or comparison with other countries | We appreciate that Australian and New Zealand data was used to illustrate MSK burden of disease, but do note that the introduction refers to the Global Burden of Disease project. We have added information from the US. | New text added to introduction: “In the United States, funding for the National Institute of Arthritis and Musculoskeletal and Skin Diseases (NIAMS) have never been more than 2% of the National Institutes of Health annual disbursement (7)”. |
| Positive comments | We appreciate the reviewer’s favourable opinion on the merits of the manuscript. |  |
